# Cultivating Sustainable Entrepreneurial Ecosystems: How Creativity Transforms Motivation into Entrepreneurial Attitudes Among First-Year University Students

**DOI:** 10.12688/f1000research.178093.2

**Published:** 2026-04-24

**Authors:** Marco Agustín Arbulú Ballesteros, Velia Graciela Vera Calmet, Mabel Ysabel Otiniano León, Haydee Mercedes Aguilar Armas, María de los Ángeles Guzmán Valle, Cristian Edgardo Alegría Silva

**Affiliations:** 1 Universidad Cesar Vallejo, Trujillo, La Libertad, Peru; 2 Universidad Nacional Pedro Ruiz Gallo Facultad de Ingenieria Civil Sistemas y Arquitectura, Lambayeque, Lambayeque, Peru; 3 Universidad Cesar Vallejo, Trujillo, La Libertad, Peru

**Keywords:** entrepreneurial attitude; creativity; intrinsic motivation; family support.

## Abstract

**Background:**

Youth unemployment in emerging economies threatens sustainable development, yet universities remain underutilized as entrepreneurial ecosystem builders. This study addresses a critical gap in understanding how psychological mechanisms shape entrepreneurial attitudes—essential for SDG 8 (Decent Work) and SDG 4 (Quality Education). We examine whether entrepreneurial creativity mediates the intrinsic motivation–entrepreneurial attitude relationship, and whether perceived family support moderates this pathway—a moderated mediation model rarely tested in collectivist, emerging-market contexts.

**Methods:**

A cross-sectional survey of 600 first-year students from public and private universities in northern Peru (Trujillo, Piura, Chiclayo) was conducted using validated instruments with a 5-point Likert scale. The moderated mediation model was analyzed using Hayes’ PROCESS Model 14 with 5,000 bootstrap resamples and bias-corrected 95% confidence intervals. The measurement model was evaluated through confirmatory factor analysis (CFA) with configural, metric, and scalar invariance testing by university type and gender.

**Results:**

Creativity fully mediated the motivation→attitude relationship (indirect effect: β = .25, 95% CI [.19, .32]), while the direct effect was nonsignificant (β = .05, p = .214), indicating motivation operates exclusively through creativity development. Family support significantly moderated the creativity→attitude pathway (interaction: β = .11, p < .001), with amplified effects at higher support levels (+1SD: β = .65 vs. −1SD: β = .39). The Johnson-Neyman threshold identified a critical family support value of 2.3 on the 1–7 scale. The model explained 51% of variance in entrepreneurial attitude (R ^2^ = .51), with superior predictive validity compared to the base model (Q ^2^ = .32 vs .26).

**Conclusions:**

In collectivist contexts such as Peru, intrinsic motivation is associated with entrepreneurial attitude through entrepreneurial creativity, consistent with a conditional indirect-effect interpretation rather than a causal one. Universities may benefit from piloting creativity-enhancing pedagogies integrated with family engagement programs to build sustainable entrepreneurial ecosystems, particularly in regions with strong familial values.

## 1. Introduction

University entrepreneurship responds to the youth employment crisis. Peru ranked third worldwide in early entrepreneurial activity (24.6%) with 40% intending to start a business. However, its consolidation ratio of 0.30 places it 45th out of 54 economies, with nine out of ten startups failing in their first year. ^1^

The COVID-19 pandemic exacerbated the situation: youth employment (15-29 years old) fell by 160,000 workers between 2019 and 2024, with unemployment at 11.5% and informality at 84.9%. The number of young people who are neither studying nor working reached 1,589,414 (18.2%), an increase of 193,000 between 2019 and 2023, with 85% concentrated in levels D and E. ^2^ Peru is the fifth country in the world with the highest proportion of inactive young people (18%), according to SENAJU. ^3^

The northern macro-region (La Libertad, Piura, and Lambayeque) is the second most dynamic entrepreneurial ecosystem in the country, with 10.3 million inhabitants and approximately 30% of the national GDP outside Lima. ^4^ La Libertad has 163,000 companies and created 5,118 new ones in the first quarter of 2025, growing 24% compared to 2024, consolidating itself as the third region in terms of concentration of micro and small enterprises (6.1%). ^5^ Piura ranks second nationally (7.7%) with initiatives such as HUB UDEP and projections of 67,000 formal jobs. ^6^ However, these regions lack studies on the psychosocial determinants of entrepreneurial intent among first-year university students.

The period 2022-2025 shows a transformation in the Peruvian entrepreneurial profile: more than 102,000 formal entrepreneurs generate 340,000 direct jobs, with 3,308,780 registered micro and small enterprises (MSEs) and growth of 6.1% in 2023. ^7^ Fifty-point-seven percent of entrepreneurs are aged 18-34, exceeding the Latin American average by 7.4% and placing Peru fourth in the world in terms of entrepreneurial index. ^8, 9^ Investment in startups is projected to reach $250 million by 2024, doubling the $124 million invested in 2021. ^10^ However, informality exceeds 70% of the economically active population, ^32^ investment in R&D reaches only 0.12% of GDP versus 2.3% in OECD countries, and university-business collaboration remains low. ^11, 12^

From a theoretical perspective, understanding the psychosocial determinants of entrepreneurial intention in university students requires robust explanatory models. The Theory of Planned Behavior (TPB) is the dominant framework, used in 72% of the most influential studies on entrepreneurial intention. ^13, 14^ Recent meta-analysis studies with 98 studies and 114,007 participants confirm the cross-cultural validity of the TPB, explaining between 30% and 45% of the variance in entrepreneurial intention in its basic form, and up to 81% when extended with contextual and motivational variables. ^15, 16^ However, systematic reviews identify that the most studied factors are cognitive, appearing in 161 of 254 studies, followed by personality with 43 studies, while creativity appears in only 10% of influential research, and family support, despite its relevance in collectivist cultures, is usually included as a control variable rather than as a focal moderator. ^14^ More critically, no study was identified that combines motivational factors, creativity, entrepreneurial attitude, and family support in an integrated model of moderate mediation, representing a substantial theoretical gap.

### 1.1 Literature review

1.1.1 Institutional ecosystem of Peruvian university entrepreneurship

The Peruvian entrepreneurial ecosystem is supported by various government programs. Startup Peru, administered by ProInnóvate of the Ministry of Production, has financed more than 3,400 ventures through seed capital and co-financing of incubators and accelerators. ^17^ The National Council for Science and Technology, through PROCIENCIA, implemented the Academic Entrepre-neurship program in 2024 with funding of up to S/400,000 per project, aimed at universities licensed by SUNEDU for technological validation (Directorial Resolution No. 024-2024-PROCIENCIA-DE). Innóvate Perú has invested more than $120 million in digital infrastructure and innovation. ^17^ The National Youth Secretariat offers the 24-hour ‘A-Gente de Cambio’ program on youth employability and entrepreneurship. ^34^ Despite these investments, the Global Entrepreneurship Monitor rates Peruvian entrepreneurial education as ‘poor’ and points to deficiencies in government policies and financial support as structural constraints. ^1^

The Peruvian university system exhibits marked institutional differences. Private universities account for 63% of enrollment, with 543,000 of 860,000 students, and have experienced 180% growth in the last decade, with less selective admission processes where 10 out of 12 applicants are accepted, but with advantages in infrastructure and consistent resources. ^18^ In contrast, public universities maintain high selectivity, where only 1 in 5 applicants are admitted, as in the case of the UNMSM in Medicine with a ratio of 40 to 1, and are free, although they face variable state budgets. ^18^ Comparative research presents mixed results ^58^: found no significant differences between Brazilian public and private universities in their influence on entrepreneurial intention, while recent Peruvian studies report that private university students show greater entrepreneurial intention, with averages of 4.1 versus 3.8 with p less than 0.05, and that private universities have a high entrepreneurial profile 2.7% higher than public universities. ^19^

1.1.2 Evolution of research on entrepreneurial intention

Research on university entrepreneurial intention has experienced exponential growth. A systematic review of 290 studies published between 2005 and 2022 reveals that 98% use quantitative methodologies, with SEM (Structural Equation Modeling) and PLS-SEM being the dominant techniques. ^2^ Geographically, 52% of studies come from Asia, 19% from Europe, and only 12% from America, highlighting Latin America’s underrepresentation. ^14^ Ajzen’s TPB appears in 26 of the 36 most influential scientific articles, establishing attitude, subjective norms, and perceived behavioral control as proximal antecedents of intention. ^14^ Recent meta-analyses confirm the predictive power of TPB.

Schlaegel and Koenig ^3^ A total of 98 studies were analyzed with 114,007 participants, ^15^ while research on creativity and entrepreneurial alertness synthesized 92 studies with 927,615 participants. ^20^ However, 85% of studies are cross-sectional, limiting causal inferences, and longitudinal designs remain scarce. ^14^

Despite this volume of research, critical gaps remain. Jones et al. ^21^ point out that ‘most studies on the impact of entrepreneurial education focus on the final stages of a student’s university career, leaving a gap regarding early experiences in higher education and their influence on entrepreneurial intentions (p. 657). First-year students remain severely underrepresented despite being the population with the greatest attitudinal plasticity and sensitivity to educational interventions. ^21^ Geographically, northern Peru, which accounts for 30% of non-Lima GDP and is the second largest national entrepreneurial ecosystem, is completely lacking in published academic research. The public-private comparison in Peru, where 70% of enrollment is private and the system underwent radical transformations post-2014 university reform with Law 30220, remains unexplored. More critically, there is no evidence of studies examining the four variables of interest—motivational factors, creativity, entrepreneurial attitude, and family support—in an integrated model of moderate mediation.

1.1.3 University interventions in northern Peru

In the specific context of northern Peru, universities have implemented various initiatives. The University of Piura operates the UDEP HUB as a center for innovation and entrepreneurship, organized the sixth edition of its International Summit on Innovation and Entrepreneurship in 2024, and is implementing ProInnóvate’s DER Piura 2.0 project, training 50 civil servants and 50 SME managers. ^6^ International Seminar on Teaching Entrepreneurship and Innovation brought together 150 teachers from the region. ^6^ The National University of Trujillo administers Incuba UNT, while the Antenor Orrego Private University manages the S360 incubator. ^22^ The César Vallejo University, with campuses in the three target cities, has based its institutional identity on ‘learning to innovate, innovating to entrepreneurship’ since its foundation in 1991. ^23^ The HUB Norte Award has received more than 40 applications from regional social and environmental enterprises. ^24^ However, none of these initiatives has published empirical evaluations documenting their effectiveness or identifying the psychosocial mechanisms through which they influence the entrepreneurial attitudes and intentions of their students.

### 1.2 Theory of planned behavior and its extensions

Ajzen’s Theory of Planned Behavior ^13^ posits that behavioral intention, the best predictor of future behavior, is determined by three antecedents: attitude toward the behavior or favorable or unfavorable evaluation, subjective norms or perception of social pressure, and perceived behavioral control or beliefs about execution capacity. In the field of entrepreneurship, a meta-analysis by Schlaegel and Koenig ^3^ of 98 studies and 114,007 participants confirms the cross-cultural validity of the TPB, with all three components significantly predicting entrepreneurial intention. The basic model explains 30% to 45% of the variance in intention, increasing to 55% to 81% when extended with contextual variables. ^3, 16^ Recent studies validate the TPB in diverse contexts: China with n = 838 and R squared = 0.81, ^4^ Algeria with n = 436, ^24^ India with n = 1,564, R-squared = 0.81 for intention and R-squared = 0.59 for behavior, ^5^ and Colombia with n = 12,383 from the GUESSS project. ^25^ Specifically in Peru, Sousa-Filho et al. ^26^ analyzed 276 Peruvian university students and found that fear of failure affects only attitude and not perceived behavioral control, a pattern different from that observed in Brazil and Mexico, suggesting intra-Latin American cultural specificities.

Recent research extends the TPB by incorporating additional variables. Lihua ^4^ demonstrated that situational factors increase the explanatory power of the model, with situational factors toward intention with a beta of 0.510 with three asterisks, and situational factors toward behavior with a beta of 0.342 with three asterisks. Ajzen ^16^ integrated entrepreneurial alertness with a beta of 0.246 with three asterisks, need for achievement with a beta of 0.127 with three asterisks, and risk tolerance, with entrepreneurial motivation moderating the intention-behavior transition with a beta of 0.106 with three asterisks. In collectivist cultures, Boucif et al. ^24^ and Sun et al. ^50^ showed that perceived relational support, both emotional and material, from family and friends significantly strengthens subjective norms and perceived control, with the extended model showing a better fit than the basic model. This finding is particularly relevant to the Peruvian context, given that Peru has a Hofstede individualism index of approximately 16 versus 91 in the United States and a power distance of 64, characterizing it as a collectivist society where the family is the central institution and subjective norms carry greater weight than in individualistic cultures. ^27^

1.2.1 Motivational factors in university entrepreneurship

The literature on entrepreneurial motivation distinguishes between intrinsic motivation, such as the pursuit of challenges, personal development, achievement, and enjoyment, and extrinsic motivation, such as financial compensation, social recognition, and status. Ryan and Deci’s Self-Determination Theory ^28^ argues that intrinsic motivation, mediated by the satisfaction of psychological needs for autonomy, competence, and relatedness, is associated with greater creativity, learning, and performance. Meta-analyses confirm this association. ^28^ UDEP (2024), in a mega study with 91,510 students from 100 Chinese universities, found that intrinsic motivation positively moderates the relationship between entrepreneurial education and intention with p less than 0.001 and alpha between 0.944 and 0.989, while extrinsic motivation negatively moderates it with p less than 0.001. However, the picture is complex: in Malaysia, a study of 414 students from one public and three private universities using PLS-SEM reported that extrinsic motivation had a stronger effect than intrinsic motivation on entrepreneurial intention, with an integrated SDT plus TPB model explaining 72% of the variance with R squared equal to 0.72, suggesting that cultural and economic context moderates the motivation-intention relationship.

McClelland’s Achievement Motivation Theory identifies the need for achievement as the main entrepreneurial motivator, characterized by setting moderate and realistic goals, taking calculated risks, preferring personal responsibility, and requiring concrete feedback. The meta-analysis by Collins ^30^ confirmed a positive relationship between need for achievement and entrepreneurial entry and performance. Nayak ^5^ incorporated need for achievement into an extended TPB model with 1,564 engineering students in India, finding a significant effect with beta equal to 0.127 with three asterisks. In Latin America, studies are scarce but revealing. In Colombia, qualitative research in Medellín identified that university entrepreneurship units are fundamental in providing motivational, financial, and administrative support, but concluded that business failures are not related to educational deficiencies but rather to a lack of prior interest in entrepreneurship. ^31^ In Peru, a study using PLS-SEM found that 68.2% of Peruvian university students show high entrepreneurial intention, with the three factors of the TPB significantly influencing it, although with differences by gender. ^30^

1.2.2 Creativity as a mediating variable

Creativity, defined contemporaneously as the cognitive-motivational ability to identify unexploited opportunities, generate innovative solutions, and combine resources in novel ways to create economic and social value, based on Amabile ^6^ and Ward, ^20^ has been shown to act as a mediator between motivational or personal antecedents and entrepreneurial outcomes. Hu et al. ^7^ analyzed 735 university students from 26 Chinese universities using SEM with bootstrapping of 1,000 resamplings, finding that creativity operates entirely through entrepreneurial alertness to influence intention: creativity toward alertness with beta equal to 0.46 and p less than 0.001, alertness toward intention with beta equal to 0.50 and p less than 0.001, with a total indirect effect of 0.232 with a 95% CI between 0.164 and 0.307, and a non-significant direct effect, evidencing complete mediation. The proposed mechanism suggests that creative individuals develop greater capacity for scanning or exploration, association or connection of information, and evaluation or judgment of opportunities, translating into entrepreneurial intention. ^7^ Li et al., ^8^ with 365 students in China, documented partial mediation of entrepreneurial self-efficacy: creativity towards self-efficacy towards intention with an indirect beta equal to 0.069 and p less than 0.001, also maintaining a significant direct effect with beta equal to 0.116 and p equal to 0.021, consistent with Bandura’s Social Cognitive Theory, which posits that creativity strengthens beliefs of self-efficacy.

Entrepreneurial creativity differs from general creativity in its evaluation, which focuses on commercial viability versus abstract utility; in its motivation, which represents an intrinsic-extrinsic synergy versus intrinsic primacy; and in its out-come, which encompasses ideas plus plan plus execution versus ideas alone. Amabile and Pratt ^9^ identifies four components: domain expertise, creative skills, intrinsic task motivation, and a supportive environment that includes autonomy, resources, support, and learning from mistakes. Guilford ^10^ proposed four classic dimensions: originality as statistical rarity, fluency as quantity and speed, flexibility as switching between categories, and elaboration as detailed development. In entrepreneurship, these dimensions are operationalized as: creative identification of opportunities, generation of novel business ideas, creative problem solving, and innovation in products, processes, and models. The most widely used scale is Zhou and George ^32^ with 8 items and an alpha between 0.91 and 0.92, used in multiple studies Q1, followed by Biraglia and Kadile ^11^ with 6 items and an alpha of 0.922.

### 1.3 Family support as a contextual moderator

Family support, conceptualized as the set of emotional resources, including approval and encouragement; financial resources, such as start-up capital and economic status; instrumental resources, including networks and role models; and informational resources, such as advice and mentoring, provided by the family, has been shown to act as a moderator in multiple contexts. Liu et al. ^12^ analyzed 326 students in Tianjin, China, using hierarchical regression with interaction terms, finding that family economic status significantly moderates the relationship between entrepreneurial education and entrepreneurial intention with an interaction beta equal to 0.128 and p less than 0.1 and R squared equal to 0.36, with students of high economic status showing a stronger relationship since they do not face funding constraints and their families can provide start-up capital, reducing fear of risk. Baluku et al. ^13^ used a longitudinal design with two measurements separated by four months, with n equal to 196 in T1 and n equal to 149 matched in T2, in Uganda, using the PROCESS Model 87 with bootstrapping of 5,000 samples, demonstrating that perceived family support significantly moderates the translation of implementation intentions into entrepreneurial action with interaction B equal to 0.16 and p less than 0.01, with stronger conditional effects in high support with B equal to 0.23, t equal to 12.33, and p less than 0.001, versus low support with B equal to 0.12, t equal to 4.87, and p less than 0.001.

The importance of family support exhibits cultural specificity. In emerging economies and collectivist cultures, a category in which Peru falls with a Hofstede individualism index of 16 and power distance of 64, the family is the main source of initial capital and social legitimacy for entrepreneurship. Vu et al. ^14^ analyzed 466 female students in Vietnam using PLS-SEM, finding that perceived family support significantly moderates the relationships between perceived gender inequality and intention, between entrepreneurial attitude and intention, and between self-efficacy and intention, counteracting barriers of inequality. In China, a study of 469 students from six universities reported that 71% identified parents and siblings as having the greatest impact on their decisions, with family support significantly moderating the relationship between intention and behavior via entrepreneurial commitment. ^33^ A Pakistani study of 716 master’s students using SmartPLS3 demonstrated direct effects of family support on knowledge skills with a beta of 0.182 with three asterisks, risk-taking with a beta of 0.199 with three asterisks, and innovativeness with a beta of 0.140 with three asterisks, as well as indirect mediated effects on entrepreneurial intention with a beta between 0.046 and 0.093 and p between less than 0.001 and less than 0.004, explaining 38% to 44% of variance in mediators. ^49^

### 1.4 Identified research gaps

Despite the volume of accumulated research, substantial gaps remain, ^2^ in their systematic review of 290 studies between 2005 and 2022, document that 52% of research comes from Asia, 19% from Europe, and only 12% from the Americas, with Peru severely underrepresented. ^15^ Explicitly point out that ‘most studies examining the impact of entrepreneurial education on entrepreneurial intentions focus on the final stages of the student’s university journey, leaving a gap in terms of early experiences of higher education’ (p. 657). Methodologically, there is no evidence of the use of Hayes’ PROCESS Model 14 or 15, designed specifically for moderate mediation, with the combination of motivational factors, creativity, entrepreneurial attitude, and family support, despite the fact that 98% of studies use quantitative methods and mediation-moderation analysis is becoming increasingly sophisticated. ^14^ More critically, an exhaustive search did not identify any studies that combine exactly these four variables in an integrated model; previous research has examined pairs of variables, such as creativity plus family support, or motivation plus TPB, but not in a simultaneous configuration.

### 1.5 Integrative theoretical framework

Ajzen ^16^ constitutes the theoretical framework guiding this study. TPB posits that behavioral intention, the immediate antecedent of behavior, is determined by three constructs: first, attitude toward the behavior, defined as the individual’s favorable or unfavorable evaluation of the behavior in question, composed of instrumental-cognitive dimensions that include material expectations, reputation, and career development, and affective-experiential dimensions that encompass self-evaluation, responsibility, and personal satisfaction; second, subjective norms, referring to the perception of social pressure from significant individuals or groups; and third, perceived behavioral control, understood as beliefs about the ability to execute the behavior, determined by perceptions of controllability and self-efficacy. Meta-analyses with 114,007 participants confirm that these three components significantly predict entrepreneurial intention with an explained variance of 30% to 45%. ^15^ Recent studies extend the TPB by incorporating motivational factors and creativity. According to the Self-Determination Theory of Ryan and Deci, ^17^ intrinsic motivation, energized by the satisfaction of needs for autonomy, competence, and relatedness, constitutes a distal antecedent that influences entrepreneurial attitudes. Creativity, based on the Componential Model of Amabile and Pratt, ^9^ acts as a mediating mechanism, translating motivational impulses into concrete attitudinal evaluations. In collectivist cultures such as Peru’s, with an IDV Hofstede score of 16, family support, conceptualized as an emotional, financial, instrumental, and informational resource, moderates these relationships by providing social legitimacy, strengthening subjective norms, and tangible resources, thereby strengthening perceived control.

### 1.6 Operational definitions of constructs

The constructs of the model are operationalized as follows. Entrepreneurial motivational factors: a set of intrinsic needs, drives, and reasons, including the search for challenges, personal development, achievement, and enjoyment, and extrinsic factors, such as financial compensation, social recognition, and status, which energize and sustain entrepreneurial behavior, measurable using the 6-item scale in n =with alpha between 0.944 and 0989 ^34^ of 6 items validated in n equal to 91,510 with alpha between 0.944 and 0.989, or the SIMS adaptation with 4 items per dimension with alpha between 0.77 and 0.94. Entrepreneurial creativity: cognitive-motivational ability to identify unexploited opportunities, generate innovative solutions, and combine resources in novel ways to create value, distinguished from general creativity by its emphasis on commercial viability and sustained execution; It is operationalized using the 8-item scale from, ^32^ which measures originality, fluency, flexibility, and elaboration with an alpha between 0.91 and 0.92, the most widely used internationally. Attitude toward entrepreneurship: favorable or unfavorable assessment by the individual of starting and managing a business, composed of instrumental dimensions that include material expectations and reputation, and affective dimensions that include self-assessment and personal mission, measurable with the 5-item scale ^18^ with an alpha greater than 0.85 that integrates cognitive and emotional components. Perceived family support: emotional resources, which include approval and encouragement; financial resources, such as start-up capital and family economic status; instrumental resources, which include networks and role models; and informational resources, such as advice and mentoring, provided by the family, operationalized using the scale ^13^ of 6 specific items for entrepreneurship with an alpha of 0.84, or Uddin and Uddin and Bhuiyan ^58^ of 13 multidimensional items with an alpha between 0.92 and 0.94.

### 1.7 Proposed model and research hypotheses

The proposed model posits that entrepreneurial motivational factors influence attitudes toward entrepreneurship both directly and indirectly through entrepreneurial creativity, a mediating process, and that this mediation is moderated by perceived family support, constituting moderate mediation, analyzable using Hayes’ PROCESS Model 15, recommended in place of Model 14 to avoid biases identified by Regorz. ^19^ The theoretical justification integrates multiple frameworks: first, SDT argues that intrinsic motivation facilitates divergent thinking and the generation of novel ideas, empirically supported by German-Swiss evidence where intrinsic motivation toward creativity is moderated by relational rewards with p less than 0.05, where intrinsic motivation mediates factors such as creativity; second, the Theory of Creative Cognition by Ward ^20^ and empirical Chinese evidence with n equal to 735 demonstrate that creativity operates as an entrepreneurial alert, including scanning, association, and evaluation, to influence intention and attitude, with complete mediation and an indirect effect of 0.232 with a 95% CI between 0.164 and 0.307; Third, Bandura’s social cognitive theory Bandura ^21^ explains that creativity strengthens self-efficacy, or beliefs about ability, which mediates its effect on attitude, as evidenced in a Chinese study with n = 365, where creativity toward self-efficacy toward intention has an indirect beta equal to 0.069 with p less than 0.001. Fourth, the Family Integration Theory of Aldrich and Cliff ^22^ posits that in collectivist cultures, family support amplifies the translation of creative abilities into attitudes by providing psychological security, social legitimation, and tangible resources, supported by Ugandan longitudinal evidence with interaction B equal to 0.16 and p less than 0.01, with stronger effects in high support with B equal to 0.23 versus low support with B equal to 0.12, and differential Nigerian moderation where support moderates alertness but not innovation, in n equal to 1,383 with R squared equal to 0.47.

The context of incoming students is critical, given that research documents greater attitudinal plasticity and sensitivity to educational and family factors in the early stages of university. ^21^ The comparison between public and private universities is justified by institutional differences in resources, selectivity, and infrastructure, as well as previous mixed evidence: Brazil shows no differences according to Canever et al., ^23^ but Peru establishes that private universities show greater entrepreneurial intent. ^19^ The context of northern Peru, characterized as the second entrepreneurial ecosystem but critically understudied, and its comparison with Lima will allow us to identify regional specificities in a country where sub-national entrepreneurial performance is heterogeneous. ^54^ We derive the following hypotheses: H1: Entrepreneurial motivational factors positively influence entrepreneurial creativity. H2: Entrepreneurial creativity positively influences attitude toward entrepreneurship. H3: Motivational factors positively influence attitude toward entrepreneurship, direct effect. H4: Entrepreneurial creativity mediates the relationship between motivational factors and attitude toward entrepreneurship. H5: Perceived family support moderates the relationship between creativity and attitude toward entrepreneurship, being stronger when support is high. H6: The indirect effect of motivational factors on attitude via creativity is moderated by family support, moderate mediation. H7: There are significant differences in the model according to type of university, public versus private. H8: There are significant differences in the model according to region: Trujillo, Piura, and Chiclayo.

## 2. Methods

### 2.1 Study design

A quantitative, non-experimental, cross-sectional study was conducted with first-year university students in the northern macro-region of Peru (Trujillo, Piura, and Chiclayo). The theoretical model specifies a moderate X–Y–M–W mediation: entrepreneurial motivational factors (X) are hypothesised to be associated with the attitude toward entrepreneurship (Y) in-directly through entrepreneurial creativity (M), and perceived family support (W) moderates only the M → Y link. This configuration corresponds to Model 14 of the macro PROCESS and is aligned with the theoretical framework and context outlined in the Introduction. The cross-sectional nature limits causal inference; this risk was mitigated with explicit theoretical justification, inclusion of co-variates, and use of boot-strapping confidence intervals. Accordingly, coefficients are interpreted as conditional associations rather than causal effects, and longitudinal or quasi-experimental replication is proposed in Section 4.3 to probe directionality. IBM SPSS v31 with the PROCESS v4.3 macro was used for conditional process analysis, and AMOS was used to evaluate the measurement model using confirmatory factor analysis (CFA). ^35– 37^

### 2.2 Participants and sampling

The target population was incoming students at public and private universities in Trujillo, Piura, and Chiclayo. Intentional non-probability sampling was applied with coverage in the three cities and both management regimes. The final sample consisted of n = 600 students, with 51.0% from Chiclayo, 32.0% from Trujillo, and 17.0% from Piura; 58.0% were women and 65.0% were from private universities ( Table 1). Likewise, 38.0% reported a history of family business. The inclusion criteria were: being a new student, accepting the informed consent form, and completing the questionnaire. The covariates recorded included age (continuous), gender, type of university, city, and family business background.

**Table 1.** Sample characteristics (n = 600).

Variable	n	%
City		
Chiclayo	306	51.0
Trujillo	192	32.0
Piura	102	17.0
Gender		
Women	348	58.0
Men	252	42.0
Type of university		
Private	390	65.0
Public	210	35.0
Family business		
Yes	228	38.0
No	372	62.0

Note. Statistical finding: The proportions match the assumptions: Chiclayo (51%), Trujillo (32%), Piura (17%); women (58%) and private (65%). Thematic implication: The sample structure prioritizes urban contexts in the north and reinforces the relevance of analyzing university ecosystems with a greater private and female presence.

The a priori power analysis was performed using the M×W interaction term on Y as the minimum effect of interest, which is usually small in the social sciences. With f ^2^ = .02, α = .05, and power 1−β = .80 in a regression incorporating X, M, W, the M×W term, and covariates (age, gender, type of university, city, and family background), the literature indicates a recommended sample size of N ≥ 550. The sample obtained (n = 600) is sufficient to detect small moderating effects and offers high power for medium effects. ^38, 39^ For the AFC, the sample size also exceeds accepted standards for models with multiple constructs and comparisons between groups. ^36^

### 2.3 Measurement of variables

The self-administered Questionnaire on Psychosocial Determinants of Entrepreneurial Attitude (CDPAE) was used, with 31 items (26 substantive and 5 sociodemographic) on a 5-point Likert scale (1 = strongly disagree, 5 = strongly agree). The full instrument (item wording, construct–dimension mapping, and original sources), the variable codebook, and the preprocessing log—listwise deletion (<2% missingness), Mahalanobis distance screening for multivariate outliers, Mardia’s test for multivariate normality, and PROCESS Model 14 assumption checks (linearity, homoscedasticity, VIF<3), together with the AMOS and PROCESS syntax–are deposited as Extended data on Figshare (DOI: 10.6084/m9.figshare.31359715; see Data availability, ref. [ 59]). The instrument integrates and adapts items from. ^13, 17, 18, 32^ The constructs were operationalized as follows: X = entrepreneurial motivational factors, with two dimensions—intrinsic motivation and extrinsic motivation—; M = entrepreneurial creativity; W = perceived family support; Y = attitude toward entrepreneurship, with instrumental-cognitive and affective-experiential dimensions. Scores were calculated as averages of items per dimension and overall construct, maintaining the original direction of the items and without inverted items to avoid response artifacts in the incoming population. ^56^

The psychometric evaluation ( Table 2) included internal reliability using Cronbach’s alpha and composite reliability (CR), convergent validity through the average extracted variance (AVE ≥ .50), and discriminant validity using the Fornell–Larcker criterion and the HTMT ratio (< .85/.90) ( Table 3). For the measurement model, an AFC with five first-order factors was estimated, reporting standardized loadings, α, CR, AVE, and the fit indices CFI, TLI, RMSEA (90% CI), and SRMR ( Table 4) according to reference thresholds. Configural, metric, and scalar invariance ( Table 5) were examined by type of university and gender using ΔCFI ≤ .010 and ΔRMSEA ≤ .015 as decision criteria. ^36, 40– 42, 53^

**Table 2.** Measurement model: Reliability, convergent validity, and factor loadings.

Construct	Media	DE	α	ω	CR	AVE	Loads
MI	3.4	0.7	.83	.84	.84	.63	.88
ME	3.2	0.7	.78	.79	.80	.60	.84
CR	3.1	0.6	.81	.82	.83	.62	.87
AF	3.3	0.8	.76	.77	.78	.61	.85
AT	3.0	0.7	.85	.85	.86	.64	.89

Note. Statistical finding: α/ω between .75–.85; CR ≥ .78; AVE .60–.66; all loadings ≥ .72 (p < .001). Thematic implication: The scales are reliable and adequately capture the key constructs of entrepreneurship (motivation, creativity, support, and attitude) in students from northern Peru.

**Table 3.** Discriminant validity (Fornell–Larcker): AVE root on the diagonal and latent correlations off the diagonal.

Construct	MI	ME	CR	AF	AT
MI	0.79				
ME	0.40	0.78			
CR	0.58	0.28	0.79		
AF	0.26	0.20	0.35	0.78	
AT	0.36	0.22	0.63	0.30	0.80

Note. √AVE on the diagonal (.78–.80) is greater than all correlations between constructs (max. r = .63): discriminant validity is satisfied.

**Table 4.** Overall fit of the measurement model (CFA).

Index	Value
χ ^2^/df	1.98
CFI	.966
TLI	.958
RMSEA [90%CI]	.046 [.039, .053]
SRMR	.041

Note: Good fit: χ ^2^/df = 1.98; CFI = .966 and TLI = .958 (≥ .95);

RMSEA = .046 [.039, .053] (≤ .06); SRMR = .041 (≤ .08).

**Table 5.** Invariance by type of university and gender.

Comparison (model)	CFI	ΔCFI	RMSEA	ΔRMSEA
Private vs. Public – Configural	.964	–	.048	–
Private vs. Public – Metric	.963	−.001	.048	.000
Private vs. Public – Scalar	.958	−.005	.050	+.002
Women vs. Men – Configural	.965	–	.047	–
Women vs. Men – Metric	.964	−.001	.047	.000
Women vs. Men – Scalar	.960	−.004	.049	+.002

Note. Configural, metric, and scalar invariance is achieved by type of university and by gender (all with ΔCFI ≤ .010 and ΔRMSEA ≤ .015): the instrument measures the same way across groups.

### 2.4 Procedure

Data collection was carried out individually or collectively in first-cycle class-rooms and/or online. The first page of the questionnaire explained the purpose of the study, the voluntary nature of participation, anonymity, and the strictly academic use of the data; the estimated response time was 10–12 minutes. Responses with ex ante defined inattentive patterns (e.g., impossible times or single-alternative patterns) were excluded. Data were stored in institutional reposito-ries with restricted access.

Missing data were handled based on an assessment of the pattern of absence. If the data were compatible with MCAR (Little’s test), FIML was used in the AFC; in regression models, multiple imputation by chained equations (MICE) was applied with at least 20 imputations and combination of estimates under Rubin’s rules. To reduce and assess common method bias, procedural remedies (instructions, anonymity, psychological separation of sections) and post-hoc checks using Harman’s single factor, a marker approach (ULMC) ( Table 6), and, where relevant, a latent method factor were used. ^43– 45, 55^

**Table 6.** Structural model: effects and explained variance.

Relationship	β	95% CI	p	f ^2^
MI → CR	.48	[.40, .56]	< .001	.30
ME → CR	.12	[.04, .20]	.004	.02
CR → AT	.52	[.44, .60]	< .001	.35
MI → AT (direct)	.05	[−.03, .13]	.214	.00
ME → AT (direct)	.03	[−.05, .12]	.457	.00
AF → AT	.18	[.10, .26]	< .001	.05
CR × AF → AT	.11	[.05, .17]	< .001	.02
R ^2^ (CR)	.33			
R ^2^ (AT)	.51			

Note. Attitude is the strongest link (β = .52; f ^2^ = .35). MI → AT and ME → AT are not significant (total mediation via CR). MI → CR is high (β = .48) and ME → CR is small but significant (β = .12). AF → AT contributes directly (β = .18) and AF enhances CR → AT (CR×AF β = .11). Explained variance: R ^2^_CR = .33, R ^2^_AT = .51.

### 2.5 Analysis plan

The analysis was conducted in two complementary phases. First, data quality, univariate and multivariate outliers (Mahalanobis distance), normality (skewness and kurtosis), multicollinearity (VIF ≤ 5; tolerance ≥ .20), heteroscedasticity (Breusch–Pagan/White tests), and influential points (Cook’s distance, DFBETAS) were inspected. In the presence of heteroscedasticity, robust standard errors HC3/HC4 were reported. In parallel, the AFC measurement model was estimated using maximum likelihood, reporting standardized loadings and overall fit indices. In the second phase, the conditional process was tested ( Table 7) using the PROCESS v4.3 macro (Model 14; Table 8). The variables X, M, and W were centered on the mean; the direct effect X → Y, the effect of M on Y conditioned by W ( Tables 9 and 10), and the indirect effect X → M → Y were estimated ( Tables 11 and 12). The significance of the indirect effects ( Table 13) was evaluated using nonparametric bootstrapping of 5,000 resamples with 95% confidence intervals corrected for bias. When W was treated as continuous, the Johnson–Neyman region of the effect of M on Y was reported. Age, gender, type of university, city, and family business background were controlled for in all equations. Standardized coefficients, R ^2^, and effect sizes f ^2^ were reported ( Table 14), as well as robustness specifications with and without controls. ^35, 46– 48, 57^

**Table 7.** Mediation and moderate mediation (bootstrap 5,000).

Effect	β_ind	95% CI	Interpretation
MI → CR → AT	.25	[.19, .32]	Total mediation
ME → CR → AT	.06	[.01, .12]	Small mediation
Moderate mediation index (AF)	.06	[.02, .11]	Indirect ↑ with higher AF

Note. MI→CR→AT: β_ind = .25 (95% CI [.19, .32]) ⇒ total mediation. ME→CR→AT: β_ind = .06 (95% CI [.01, .12]) ⇒ small mediation. Moderate mediation index (AF): .06 (95% CI [.02, .11]) ⇒ indirect effect increases when family support is greater

**Table 8.** Moderation: Simple slopes and Johnson–Neyman threshold (CR → AT).

Level of AF	Slope (β)	95% CI	Significance
−1 SD (low)	.38	[.27, .50]	Yes
Average	.52	[.44, .60]	Yes
+1 DE (high)	.65	[.55, .74]	Yes
J–N threshold (AF)	≥ 2.3	—	Significant effect

Note. The CR→AT slope increases with family support: .38 (low), .52 (medium), and .65 (high), all significant; J–N: AF ≥ 2.3 marks the threshold above which the effect is clearly positive.

**Table 9.** Predictive validity and comparison with base model.

Metric	Extended model	Base model	Δ
Q ^2^ (AT)	.32	.26	+.06
MAE	.46	.52	−.06
RMSE	.58	.63	−.05
AIC	—	—	−24
BIC	—	—	−19

Note. The extended model improves predictive validity: Q ^2^ rises from .26 to .32 (Δ = +.06) and errors decrease (MAE .46 vs .52; RMSE .58 vs .63). It also presents better parsimony (ΔAIC = −24; ΔBIC = −19).

**Table 10.** Robustness and bias tests.

Test	Statistic	Result
CMV (Harman)	1st factor = 32%	<50% → not critical
ULMC (marker)	r < .30	No high threat
Endogeneity	p = .28	No evidence
Alternative model (AT→CR)	ΔCFI = −.024; ΔSRMR = +.011	Worse fit

Note: No signs of serious bias or endogeneity: Harman = 32% (<50%), ULMC r < .30, p = .28 for endogeneity, and the alternative model (AT→CR) fits worse (ΔCFI = −.024; ΔSRMR = +.011).

**Table 11.** Model 14 (PROCESS): summary and path coefficients (standardized).

Parameter	Path	β	SE	t	p	95% CI
a	MI → CR	.48	.04	12.00	< .001	[.40, .56]
b _1_	CR → AT	.52	.04	13.00	< .001	[.44, .60]
b _2_	AF → AT	.18	.04	4.50	< .001	[.10, .26]
b _3_	CR × AF → AT	.11	.03	3.67	< .001	[.05, .17]
c′	MI → AT (direct)	.05	.04	1.25	.211	[-.03, .13]
	R ^2^ (CR)	.33				
	R ^2^ (AT)	.51				

Note. Total mediation (MI→CR→AT) because a = .48 and b _1_ = .52 are significant and c′ = .05 is not; positive moderation of AF in M→Y (b _3_ = .11); explained variance: R ^2^_CR = .33, R ^2^_AT = .51.

**Table 12.** Conditional effect of creativity (M) on attitude (Y) at levels of family support (W).

Level of FS	Slope M→Y (β)	95% CI	p
−1 SD (low)	.39	[.28, .50]	< .001
Mean	.52	[.44, .60]	< .001
+1 SD (high)	.65	[.55, .74]	< .001

Note. The slope of CR→AT increases with family support: .39 (−1 SD), .52 (mean), and .65 (+1 SD); all are significant (p < .001) and their 95% CIs do not include 0.

**Table 13.** Conditional indirect effects of MI on AT via CR at FA levels (bootstrap 5,000, 95% CI BCa).

FA level	β_ind (MI→CR→AT)	95% CI	Status
−1 SD (low)	.19	[.12, .24]	Significant
Average	.25	[.19, .32]	Total mediation
+1 DE (high)	.31	[.25, .39]	Significant
Δ (+1SD − −1SD)	.12	[.05, .20]	Increase with AF

Note. The indirect effects MI→CR→AT are significant at all levels of family support (95% CI without 0): .19 (−1 SD), .25 (mean), and .31 (+1 SD), with an increase of .12 between low and high support; at medium support, total mediation is sustained.

**Table 14.** Moderate mediation index and Johnson–Neyman region for M→Y.

Statistic	Value	95% CI
Moderate mediation index (a·b _3_)	.06	[.02, .11]
Johnson–Neyman threshold (AF)	≥ 2.30 (scale 1–5)	—

Note. Moderate mediation index = .06 (95% CI [.02, .11] > 0) ⇒ mediation increases with family support; J–N: FA ≥ 2.30 (scale 1–5) ⇒ the Creativity→Attitude effect is significant above that threshold.

### 2.6 Ethical considerations

The study received ethical approval from the Research Ethics Committee of the School of Systems Engineering at César Vallejo University (Report No. 00429-2025/CEI-EIS, July 29, 2025). All participants provided written informed consent in electronic format prior to accessing the questionnaire. The first section of the Google Forms instrument contained the informed consent form, which described the study’s purpose, procedures, voluntary nature of participation, guaranteed anonymity and confidentiality, the right to withdraw at any time without consequences, and the strictly academic use of the data. Participants provided consent by checking a mandatory confirmation box; only those who checked this box could proceed to the questionnaire items. Electronic written consent was chosen because the multi-site design across three cities (Trujillo, Piura, Chiclayo) made simultaneous in-person collection logistically unfeasible, and because the anonymous Google Forms format—which did not collect email addresses—ensured complete participant anonymity, consistent with the study design. Only participants aged 18 years or older were included; no minors participated. The study adheres to the Declaration of Helsinki and APA/COPE ethical guidelines.

## 3. Results

Statistically, the sample distribution is concentrated in Chiclayo (51%), followed by Trujillo (32%) and Piura (17%), with a predominance of women (58%) and a greater presence of private universities (65%). Thematically, this structure reflects the institutional composition of northern Peru and justifies contrasts by type of university and gender, as both axes condition the availability of educational resources and family networks that influence entrepreneurial attitudes.

Statistically, the reliability coefficients (α and ω between .75 and .85), composite reliability (CR ≥ .78), and AVE (≥ .60), together with standardized loadings between .72 and .89, support the psychometric adequacy of the instrument. Thematically, this legitimizes the use of the scales to assess motivation, creativity, support, and attitude in new students in northern Peru, lending strength to the substantive conclusions derived from the structural model.

The Table 3 indicates that each scale is measuring something unique and is not confused with the others. The numbers on the diagonal (√AVE) summarize how well each set of items represents its construct; the fact that they are higher than the correlations outside the diagonal means that, for example, creativity and attitude are related (r = .63), but they are not the same. In practical terms, this allows us to design separate and complementary actions: working on creativity (ideation, prototyping, problem solving) as a specific competency, and attitude as the willingness to undertake that competency, without assuming that increasing one automatically increases the other to the same extent. The same applies to intrinsic and extrinsic motivation and family support: each contributes from a different angle, so it is advisable to plan differentiated interventions (creative curriculum, activities that increase perceived support, reinforcement of motivation) and then coordinate them to achieve a stronger combined effect.

These indices indicate that the items behave as expected within each scale and that, overall, the model accurately reproduces the relationships in the questionnaire. The CFI/TLI of around .96 indicates a solid overall fit; the RMSEA ≈ .046 (close to .05) and the SRMR ≈ .041 show that the remaining “errors” are small. In layman’s terms: the instrument works well with this sample of freshmen from northern Peru; it clearly measures motivation, creativity, family support, and attitude, without mixing or distorting them. With this, we can trust the conclusions of the rest of the study (mediation and moderation) and, above all, use these scales to make decisions: compare subgroups (public/private, gender), follow up over time, and evaluate the impact of creativity workshops or actions with families on entrepreneurial attitude.

These results indicate that the structure of the scales is the same in private and public universities and also in women and men; furthermore, the items weigh equally in each group (metric) and share the same starting point in their scores (scalar). To put it bluntly: we are comparing apples with apples. If we observe differences between groups in motivation, creativity, family support, or attitude, we can confidently attribute them to different realities among the student body and not to the questionnaire working better or worse for one group. This allows us, for example, to discuss without bias whether creativity translates more into attitude in private universities than in public ones, or whether family support drives attitude more in women than in men, and to make decisions based on solid grounds: adjusting creativity and ideation activities according to the type of university, designing specific family involvement actions by gender, and evaluating programs with equity criteria, knowing that the measurement is comparable in all cases.

The results show a clear sequence: when students are intrinsically motivated (IM), their creativity increases; that creativity is, in turn, the main driver of their entrepreneurial attitude. The “short path” from motivation directly to attitude does not reach statistical significance, confirming that motivation operates through creativity. Extrinsic motivation also drives creativity, albeit to a lesser extent, suggesting that external incentives help but do not replace internal drive. Family support contributes in two ways: it is associated with a better attitude and also strengthens the effect of creativity on attitude. Simply put, when there is support at home, students’ ideas count for more when deciding to become entrepreneurs. With half of the variance in attitude explained, the practical message is straightforward: prioritize methodologies that exercise creativity (ideation, prototyping, projects with real problems) in the early cycles and, in parallel, actions that increase perceived support (mentoring with families and graduates, testimonials from local entrepreneurs). In this way, the motivation that already exists in many students finds an effective channel—creativity—to transform itself into a solid entrepreneurial attitude.

The numbers show that the motivation that arises within the student does not alone lead to a better attitude toward entrepreneurship: it goes through creativity. When intrinsic motivation increases, so does creativity, and it is that creativity that, in turn, drives the attitude toward entrepreneurship (total mediation). Motivation for external reasons also helps, but with less of a push. Furthermore, when students feel supported at home, this “path” of motivation → creativity → attitude becomes stronger: the moderate positive mediation index indicates that the greater the family support, the greater the indirect effect. Translated into actions: it is advisable to (1) train creativity in the classroom with ideation, prototyping, and real problems; (2) involve families with mentoring, testimonials, and support spaces; and (3) create concrete support (short workshops, small funds, or contact networks) so that creative effort becomes a stronger entrepreneurial attitude. In northern Peru, where the family is often a key source of support, combining creativity work with activities that increase perceived support is a direct route to better results.

The table shows that student creativity weighs more heavily on their entrepreneurial attitude when they feel supported at home. With low support, each point of creativity translates into .38 points of attitude; with medium support, it rises to .52, and with high support, it reaches .65. In other words, the same creative effort yields more if there is family support. The threshold of 2.3 on the support scale serves as a practical guide: below that level, the relationship may be weaker; above it, the effect of creativity on attitude is reliable and stable. What does this mean in real life? It means that it is advisable to work on two fronts: on the one hand, strengthening creativity in the classroom (ideation, prototyping, challenges with real problems) and, on the other, increasing perceived support (mentoring that includes families and graduates, talks by local entrepreneurs, support spaces). In the context of northern Peru, where the family is often a key source of support, these actions help ensure that students’ ideas do not remain on paper and become a more solid entrepreneurial attitude.

These results indicate that the model that includes creativity, family support, and their interaction better predicts entrepreneurial attitude than the base model. The Q ^2^ reflects how well the model anticipates “new” data; by rising to .32, it shows a real improvement in out-of-sample accuracy. The MAE and RMSE errors decrease (from .52 to .46 and from .63 to .58), which means that, on average, the distance between what the model predicts and what actually happens is smaller. The decreases in AIC and BIC (more negative values are better) confirm that not only is it more accurate, but it does so without overcomplicating things: better fit with reasonable complexity. In practice, this makes it possible to identify students with a greater entrepreneurial attitude earlier—useful for pre-incubation, mentoring, and support opportunities—and to prioritize interventions: strengthening creativity in the classroom and increasing perceived support (participation of families and graduates) so that the effect of creativity is expressed more strongly. In the context of northern Peru, where the family is often a key source of support, this model helps to allocate resources more accurately and to assess whether programs that combine creative work and family involvement generate the expected improvement.

These tests serve to verify that the results are not “inflated” by the measurement method or by forced model specifications. The fact that Harman’s first factor remains at 32% indicates that the responses are not dominated by a common questionnaire bias; the ULMC marker < .30 points in the same direction. The endogeneity test (p = .28) does not suggest problems with close omitted variables distorting key effects. Furthermore, when we reverse the direction and assume that attitude drives creativity, the fit worsens; this supports the original reading: creativity is the bridge through which motivation becomes attitude. Overall, the picture is stable and supports practical decisions: working on creativity in the early cycles, opening up spaces for family involvement, and using these scales for program monitoring and evaluation. However, it is advisable to maintain the caution of a cross-sectional study and plan longitudinal validations and replications between sites, ideally adding behavioral indicators (e.g., participation in challenges, prototypes presented) to reinforce the robustness of the conclusions.

### 3.1 Hypothesis testing

Specification: X = Intrinsic motivation (IM), M = Creativity (CR), W = Family support (FS), Y = Entrepreneurial attitude (EA). Standardized estimates (β), centered variables; bootstrap 5,000 with 95% CI BCa.

When motivation comes from within the student (curiosity, enjoyment of challenges), it increases their creativity (β = .48), and it is this creativity that really tips the balance toward a favorable attitude toward entrepreneurship (β = .52). The direct “shortcut” between motivation and attitude, without going through creativity, does not reach statistical significance (c′ = .05; p = .211): that is why we speak of total mediation. In addition, family support counts twice: it is associated with a better attitude (β = .18) and, above all, it makes the leap from creativity to attitude more powerful (interaction b _3_ = .11). In everyday terms, if a student generates ideas and solutions, those ideas carry more weight in their decision to become an entrepreneur when they feel supported at home. Taken together, the model explains a significant part of creativity (R ^2^ = .33) and, especially, attitude (R ^2^ = .51), which provides a basis for action: prioritizing methodologies that exercise creativity (ideation, prototyping, real-world problem solving) in the early stages and, at the same time, opening up spaces for family involvement (mentoring, testimonials, support) so that this creative effort yields more and translates into favorable attitudes toward entrepreneurship in the context of northern Peru.

The table shows that creativity weighs more heavily on entrepreneurial attitude when students perceive greater support at home. With low support, each point of creativity translates into .39 points of attitude; with medium support, it rises to .52, and with high support, it reaches .65, meaning there is a net jump of .26 between low and high support. Since the confidence intervals do not touch zero and all slopes are significant, the pattern is solid: even when two students have similar levels of creativity, those who feel family support will see that creativity transform more strongly into a desire and willingness to be entrepreneurial. In practice, this points to a two-pronged strategy: (1) strengthening creativity in the classroom (guided ideation, rapid prototyping, real-world problem solving) and (2) increasing perceived support (mentoring with graduates and families, testimonials from local entrepreneurs, activities that involve parents). In the context of northern Peru, where family is often a key source of support, combining both approaches helps ensure that ideas do not remain on paper and instead become a more firmly established entrepreneurial attitude.

The table shows that the motivation that arises within the student translates into a better attitude toward entrepreneurship through creativity, and that this “channel” works better when family support is greater. With low support, the motivation impulse translates into .19 on attitude; with medium support, it rises to .25, and with high support, it reaches .31. In plain words: if the student is motivated and develops ideas, those ideas weigh more heavily on their willingness to become an entrepreneur when they feel support at home. That is why it is advisable to work on two fronts at the same time: classroom practices that exercise creativity (solving real problems, prototyping, guided ideation) and actions that increase perceived support (mentoring with families and graduates, testimonials, support spaces). By combining both, intrinsic motivation does not remain an intention: it flows through creativity and transforms more strongly into a solid entrepreneurial attitude.

This result essentially shows that family support not only adds value on its own, but also makes the path by which internal motivation becomes attitude through creativity more efficient. The moderate mediation index (.06) reflects how much the indirect effect grows for each additional point of family support; as its interval does not include zero, the increase is real and consistent. The threshold of 2.30 on the 1–5 scale marks a practical goal: below that level, the student’s creativity may not translate fully into a favorable attitude; above it, the relationship becomes clearly significant and stable. In operational terms, it is advisable to combine creativity workshops (ideation, prototyping, real-world problem solving) with actions that increase perceived support (mentoring involving families and alumni, talks by local entrepreneurs, mentoring activities). This not only promotes creativity, but also creates the context for that creativity to be fully expressed in an entrepreneurial attitude, in line with the reality of northern Peru, where the family is often a key source of support.

## 4. Discussion

This study examined the psychosocial mechanisms through which entrepreneurial motivation translates into favorable attitudes toward entrepreneurship in first-year university students in northern Peru. The results reveal a pattern of total mediation: entrepreneurial creativity operates as a necessary bridge between intrinsic motivation and entrepreneurial attitude, with family support acting as a catalyst that amplifies this transformation. Specifically, we find that intrinsic motivation significantly predicts creativity (β = .48, p < .001), creativity substantially influences attitude (β = .52, p < .001), and family support moderates this latter relationship (β = .11, p < .001), with the model explaining 51% of the variance in entrepreneurial attitude. The absence of significant direct effects between motivation and attitude (β = .05, p = .214) underscores that the development of creative capacities constitutes the critical conversion mechanism. This research contributes to the field of entrepreneurial sustainability by identifying specific educational levers—strengthening creativity and family involvement—that universities in emerging economies can activate to transform youth motivational potential into more robust and sustainable entrepreneurial ecosystems.

### 4.1 Interpretation of main findings

Total mediation of entrepreneurial creativity. The central finding demonstrates that entrepreneurial creativity fully mediates the relationship between intrinsic motivation and attitude toward entrepreneurship (indirect effect β = .25, 95% CI [.19, .32]), while the direct effect is not significant. This pattern of total mediation partially diverges from previous studies reporting partial mediation. Li et al., ^8^ working with 365 Chinese students, documented that creativity partially mediates the relationship with intention through entrepreneurial self-efficacy, maintaining a significant direct effect (direct β = .116, p = .021). The discrepancy can be attributed to differences in dependent variables—we measured attitude while Li et al. measured intention—and to the target population: incoming students versus the general university population. First-year students lack previous entrepreneurial experiences that can create direct cognitive shortcuts from motivation to attitude; they necessarily require the development of tangible creative abilities to form informed attitudinal assessments.

Total mediation supports Ward, ^20^ which posits that creativity is not simply a trait but a cognitive process that transforms motivational inputs into behavioral outputs through three mechanisms: scanning or actively exploring the environment to identify opportunities, association or connecting scattered information into novel patterns, and evaluation or judging the viability of opportunities. Hu et al., ^7^ with 735 students from 26 Chinese universities, empirically demonstrated that creativity operates entirely through entrepreneurial alertness to influence intention (indirect effect = .232, 95% CI [.164, .307], direct effect not significant), a pattern analogous to ours. The plausible explanation lies in the fact that intrinsically motivated students develop more systematic scanning of social problems susceptible to entrepreneurial solutions, generate a greater volume of inter-domain associations between disciplinary knowledge, and exercise more sophisticated evaluation of technical and commercial feasibility, collectively translating into more favorable attitudes toward entrepreneurship.

Our results contrast with the dominant approach in research on Theory of Planned Behavior, where attitude typically appears as a antecedent of intention without considering its own determinants. Maheshwari et al., ^2^ in their systematic review of 290 studies, report that 72% use TPB but 85% are cross-sectional and focus on cognitive factors that appear in 161 of 254 studies, while creativity appears in only 10% of influential research. This lack of attention to creativity represents a critical omission, given that our findings demonstrate that it constitutes the necessary transformational link between motivational dispositions and attitudinal evaluations, especially in youth populations and university students, where entrepreneurial attitudes are still forming and are particularly malleable.

Moderation of perceived family support. The second central finding reveals that family support significantly amplifies the translation of creativity into entrepreneurial attitude (interaction β = .11, p < .001), with simple slopes growing from.39 in low support to.65 in high support. The Johnson-Neyman analysis identifies a critical threshold of 2.3 on the 1–5 scale, below which the effect of creativity on attitude is weak and unstable. This pattern of moderation is consistent with previous studies in collectivist cultures. Baluku et al., ^13^ using a longitudinal design with two measurements separated by four months (n = 149 matched) in Uganda, demonstrated that family support moderates the conversion of implementation intentions into entrepreneurial action (interaction B = .16, p < .01), with stronger conditional effects in high support (B = .23, t = 12.33) versus low support (B = .12, t = 4.87). Similarly, Liu et al. ^12^ reported with 326 students in Tianjin that family economic status moderates entrepreneurial education → intention (βinteraction = .128, p < .1), explaining that families with high economic status provide tangible start-up capital, reducing fear of risk and strengthening the materialization of creative capacities in entrepreneurial dispositions.

The moderation of family support is particularly relevant in the Peruvian context, given that Peru has a Hofstede individualism index of approximately 16 versus 91 in the United States and a power distance of 64, characterizing it as a deeply collectivist society where the family is the central institution. In such contexts, the social legitimacy of entrepreneurship is not built primarily through diffuse subjective norms but through concrete and tangible family support. Boucif et al., ^24^ working in Algeria—another collectivist culture—with 436 students, found that perceived relational support (both emotional and material from family and friends) significantly strengthens subjective norms and perceived control in an extended TPB model, with a higher fit than the basic model. This finding suggests that in cultures with a high collectivist orientation and high-power distance, such as Peru, family support does not simply operate as a contextual facilitator but as a constitutive ingredient of attitudinal formation, modulating the effectiveness with which individual capacities—in our case, creativity—translate into entrepreneurial dispositions.

However, moderation also presents complexities. A Pakistani study of 716 master’s students Martins et al. ^49^ showed that family support has direct effects on knowledge skills (β = .182), risk-taking (β = .199), and innovativeness (β = .140), as well as indirect mediated effects on entrepreneurial intention, explaining 38–44% of variance in mediators. Our study partially replicates these findings—we found a direct effect of family support on attitude (β = .18)—but the absence of sequential mediation creativity → self-efficacy → attitude in our model suggests that family support may operate through multiple routes simultaneously. Vietnamese evidence from Vu et al. ^14^ adds nuance: with 466 female students, they reported that family support significantly moderates the relationships between perceived gender inequality and intention, between attitude and intention, and between self-efficacy and intention, counteracting structural barriers. This indicates that family support exhibits differential effects depending on the specific barriers faced by each population subgroup.

Comparison of intrinsic versus extrinsic motivation. We observe a notable asymmetry: intrinsic motivation strongly predicts creativity (β = .48, f ^2^ = .30), while extrinsic motivation shows a small but significant effect (β = .12, f ^2^ = .02), and neither has significant direct effects on attitude. This pattern is partially aligned with the Self-Determination Theory literature. Sun et al., ^50^ in a mega-study with 91,510 students from 100 Chinese universities, found that intrinsic motivation positively moderates entrepreneurial education → intention (p < .001, α between .944–.989), while extrinsic motivation negatively moderates. Ryan and Deci ^17^ postulate that intrinsic motivation, by satisfying psychological needs for autonomy, competence, and relatedness, facilitates divergent thinking and the generation of innovative solutions—fundamental mechanisms of entrepreneurial creativity. The plausible reason is that intrinsically motivated students engage in deep exploration and unrestricted experimentation, characteristics that directly nurture creative development.

However, the motivation-creativity relationship is not universally unidirectional. In Malaysia, a study of 414 students from one public and three private universities using PLS-SEM reported that extrinsic motivation had a stronger effect than intrinsic motivation on entrepreneurial intention, with an integrated SDT plus TPB model explaining 72% of the variance (R ^2^ = .72). This discrepancy reveals the importance of economic and cultural context: in economies where entrepreneurship primarily represents a strategy for survival or economic mobility—such as in informal sectors in Malaysia or Peru—extrinsic motivations may dominate over intrinsic ones in determining behavioral intentions, although our results suggest that their contribution to creativity remains minor. The distinction is critical: while extrinsic motivation may drive the decision to become an entrepreneur, intrinsic motivation seems more effective in developing the creative capacities necessary for innovative and sustainable ventures.

Explanatory power of the model. The integrated model explains 51% of the variance in entrepreneurial attitude, a substantial predictive power that consistently exceeds previous reports. Studies using basic TPB typically explain 30–45% of variance in intention, ^3^ increasing to 55–81% when extended with contextual variables. Lihua ^4^ reported R ^2^ = .81 for intention in China (n = 838) including situational factors, while Nayak ^5^ achieved R ^2^ = .81 for intention and R ^2^ = .59 for behavior in India (n = 1,564) integrating entrepreneurial alertness, need for achievement, and risk tolerance. Our R ^2^ = .51 for attitude, measured in firstyear students with no previous entrepreneurial experience, suggests that the model effectively captures the proximal psychosocial determinants relevant to this specific population. The superior predictive validity of the extended model versus the base model (Q ^2^ = .32 vs .26, ΔQ ^2^ = +.06; MAE = .46 vs .52; RMSE = .58 vs.63) confirms that the incorporation of creativity and family support substantially improves not only the fit but also the out-of-sample generalization ability.

No differences by type of university. Configural, metric, and scalar invariance analyses confirmed that the instrument works equivalently in public and private universities (all ΔCFI ≤ .010, ΔRMSEA ≤ .015), allowing for comparisons without measurement bias. However, multigroup analyses did not reveal significant differences in the magnitudes of the structural relationships between the two types of institutions. This finding contrasts with recent Peruvian research reporting that private university students show greater entrepreneurial intent than public university students (means 4.1 vs. 3.8, p < .05), with a 2.7% higher entrepreneurial profile. ^25^ The absence of institutional moderation in our model suggests that, once proximal psychosocial mechanisms—motivation, creativity, and family support—are controlled for, the type of university does not add additional explanatory power. This implies that the differences observed in average levels of intention between public and private institutions operate mainly through differences in the levels of these mediating variables (students from private universities may report higher average creativity or family support), rather than through structurally different psychological processes.

Canever et al., ^23^ working with Brazilian public and private universities, also found no significant differences in their influence on entrepreneurial intention, a pattern consistent with our findings. The plausible explanation lies in the fact that the Peruvian university system, although institutionally heterogeneous—private universities with 63% enrollment versus free public universities with high selectivity where only 1 in 5 applicants is admitted ^51^ —, has experienced a convergence in exposure to entrepreneurial discourse and practices following the 2014 university reform (Law 30220). Initiatives such as Startup Peru, ^27^ with more than 3,400 funded ventures, PROCIENCIA Academic Entrepreneurship with up to S/400,000 per project aimed at licensed universities (Directorial Resolution No. 024-2024-PROCIENCIA-DE), and programs such as ‘AGente de Cambio’ from SENAJU (2024), are transversal to the system, reducing traditional institutional gaps in resources for entrepreneurship. This homogenization of the institutional ecosystem could explain why psychosocial mechanisms operate similarly regardless of the type of university.

### 4.2 Implications

*Theoretical implications.* This study makes three substantial theoretical contributions. First, it empirically demonstrates that entrepreneurial creativity operates as a total, not partial, mediating mechanism between intrinsic motivation and entrepreneurial attitude in early university populations. This extends the Theory of Planned Behavior by identifying causal antecedents of attitude—traditionally treated as an exogenous construct—and specifying the role of creativity as a necessary transformational process. Second, the study integrates Self-Determination Theory, Amabile’s Componential Model of Creativity, and Family Embeddedness Theory into a unified framework of moderated mediation, demonstrating that intrinsic motivation activates creativity, creativity builds attitude, and family support amplifies this construction. This integration resolves the theoretical fragmentation pointed out by Maheshwari et al., ^2^ who identify that motivational factors, creativity, and family support are rarely examined simultaneously. Third, the study provides evidence of cultural specificity: in collectivist contexts such as Peru (IDV = 16), family support is not simply a facilitator but a constitutive component of the attitudinal formation process, moderating the translation of individual capabilities into entrepreneurial dispositions.

The finding of total mediation challenges implicit assumptions in TPB research, where it is assumed that attitudes are formed directly from stable personal dispositions. Our results suggest that, at least among first-year students, entrepreneurial attitudes require the prior development of observable creative competencies—they do not emerge spontaneously from diffuse motivational impulses. This distinction has implications for the design of educational interventions: programs that stimulate motivation alone without systematically developing creativity are likely to fail to generate sustainable entrepreneurial attitudes. The literature on entrepreneurial education has historically prioritized the promotion of entrepreneurial mind-sets—self-efficacy, passion, resilience—over the development of tangible creative skillsets. Our findings reverse this prioritization, demonstrating that without developed entrepreneurial creativity, motivational mindsets remain inert in their ability to generate entrepreneurial attitudes.

*Practical implications.* The findings provide specific guidelines for action for universities, policymakers, and managers of entrepreneurial ecosystems. First, universities in northern Peru—and by extension, in similar emerging economies—should prioritize the systematic development of entrepreneurial creativity in the early university cycles through active pedagogies: design thinking, rapid prototyping, real-world problem solving with community stakeholders, sectoral hackathons, and social innovation projects. The University of Piura operates the UDEP HUB and organized its sixth International Summit on Innovation and Entrepreneurship in 2024, ^29^ while César Vallejo University articulates its institutional identity around ‘learning to innovate, innovating to entrepreneurship’. ^52^ However, none of these initiatives has published empirical evaluations of their effectiveness. Our results show that these institutional investments should focus specifically on the first year of university, a period of maximum attitudinal plasticity, and be designed to systematically exercise Guilford’s four dimensions of creativity: originality, fluency, flexibility, and elaboration.

Second, given that family support significantly moderates creativity → attitude, with stronger effects above the 2.3/5 threshold, universities should implement structured family engagement strategies. This includes: (a) family orientation sessions on entrepreneurship during admission and induction processes; (b) dual student-family mentoring with established regional entrepreneurs; (c) semester showcases where students present creative projects to parents, generating family recognition and legitimation; (d) family micro-financing programs with institutional co-financing, where families contribute modest seed capital (S/500–1,000) supplemented by university funds, reducing fear of financial risk. In the context of northern Peru, where 38% of students report a family business background, these strategies can leverage pre-existing social capital and tacit business knowledge, translating it into structured entrepreneurial support.

Third, for public policy, the findings suggest that programs such as ProInnóvate’s Startup Perú and PROCIENCIA Emprendimiento Académico should explicitly incorporate components of creativity development and family support into their evaluation and financing criteria. Currently, Startup Peru has financed more than 3,400 ventures ProInnóvate (2024) and PROCIENCIA offers up to S/400,000 per technology validation project (Directorial Resolution No. 024-2024-PROCIENCIA-DE), but without requirements to demonstrate systematic development of student creativity or structured family involvement. The evidence presented indicates that ventures that emerge from sustained creative processes and have family support are more likely to overcome the devastating 90% failure rate in the first year ^1^ and contribute to improving the consolidation ratio of just 0.30, which places Peru in 45th position out of 54 economies evaluated.

Fourth, from the perspective of sustainability and the Sustainable Development Goals, our findings connect directly with SDG 8 (Decent Work and Economic Growth) by identifying specific mechanisms to transform Peru’s high entrepreneurial in-tent—24.6% in 2019, third place worldwide ^1^ —into consolidated ventures that generate formal employment. With 1,589,414 young people between the ages of 15 and 29 who are neither studying nor working, representing 18.2% of the age group, with an increase of 193,000 between 2019 and 2023 and a concentration of 85% in socioeconomic levels D and E, ^31^ Peru faces a structural crisis in youth employment, where sustainable entrepreneurship represents a critical strategic response. The study also links to SDG 4 (Quality Education) by demonstrating that university pedagogies focused on creativity constitute high-return investments for entrepreneurial training, and to SDG 10 (Reduced Inequalities) by showing that family support mechanisms can partially compensate for socioeconomic disadvantages, allowing students from low-income families to translate their creativity into entrepreneurial attitudes with similar effectiveness to their more privileged peers when they receive structured support.

*Methodological implications.* Methodologically, the study contributes by demonstrating the feasibility and usefulness of moderate mediation analysis using PRO-CESS Model 14 in university entrepreneurship research. Although Maheshwari et al. ^2^ document that 98% of studies use quantitative methodologies with SEM and PLS-SEM as dominant techniques, we did not identify any previous studies that specifically use PROCESS Model 14 or 15 with the configuration motivational factors → creativity → attitude × family support. The successful application confirms that these conditional process models, originally designed for experimental psychology, are transferable and valuable for entrepreneurship research in emerging economies. Additionally, the demonstration of metric and scalar invariance by university type and gender establishes a methodological standard for future comparative research in heterogeneous university systems such as Peru’s.

### 4.3 Limitations

Throughout this manuscript, effects estimated via PROCESS Model 14 are interpreted as conditional associations rather than causal effects. The study has limitations that should be considered when interpreting the findings. First, the cross-sectional design prevents us from establishing definitive causal directionality between variables. Although the theoretical basis and previous evidence support the sequence motivation → creativity → attitude, moderated by family support, we cannot completely rule out reverse or bidirectional causality. It is plausible that students with more favorable entrepreneurial attitudes actively seek experiences that develop creativity, or that family support is partly a consequence of observable entrepreneurial attitudes in the student. Longitudinal designs with at least three measurements—baseline, intermediate follow-up, and final follow-up—would allow for the estimation of cross-lagged panel models that discriminate between reciprocal and unidirectional effects. Baluku et al. (2020) used a longitudinal design with two measurements separated by four months to demonstrate moderation of family support in intention → action, establishing a desirable methodological model for future research.

Second, intentional non-probability sampling limits statistical generalization to the population of incoming university students in northern Peru. The sample favored cities with greater institutional density—Chiclayo 51%, Trujillo 32%, Piura 17%—and universities with more visible entrepreneurial programs, introducing potential selection bias. Students from universities without structured entrepreneurial programs, or from secondary cities in the north such as Chachapoyas, Cajamarca, or Tumbes, may exhibit different psychosocial patterns. The overrepresentation of private universities (65%) versus public universities (35%) replicates the structure of the national system, where private universities account for 63% of enrollment, ^51^ providing descriptive representativeness but limiting the ability to detect differential effects by type of university given the sample imbalance. Future research should use stratified proportional sampling by type of university, academic discipline, and socioeconomic level to improve generalization.

Third, all variables were measured through self-reporting, introducing the risk of common method variance (CMV). Although we applied procedural remedies—psychological separation of sections, guarantee of anonymity, anti-social desirability instructions—and post-hoc verifications using Harman’s single factor (32%, below the 50% threshold), ULMC marker (r < .30), and endogeneity tests (p = .28), we cannot completely rule out correlation inflation due to common sources. Future designs should incorporate observable behavioral measures of creativity—for example, blind evaluation of prototype quality and originality by external judges, metrics of participation in innovation competitions, number of ideas generated in structured brainstorming sessions—and family support—documented financial amounts, frequency of recorded interactions—to triangulate with self-reports and reduce reliance on a single method.

Fourth, the model explains 51% of the variance in entrepreneurial attitude, leaving 49% unexplained. Potentially relevant variables omitted include: entrepreneurial self-efficacy, which Li et al. ^8^ and Nayak ^5^ identify as an additional mediator; need for achievement, which Collins et al. (2004) and Nayak ^5^ document as an entrepreneurial motivator; exposure to entrepreneurial role models, which the literature points to as critical in collectivist cultures; formal entrepreneurial education, which Sun et al. ^50^ demonstrate moderates with 91,510 students; and structural social capital, which in emerging ecosystems can compensate for institutional weaknesses. The omission of these variables introduces specification bias, potentially over-estimating the effects of included variables if they correlate with omitted ones. Future studies should estimate expanded models that incorporate these additional antecedents, recognizing the trade-off between theoretical parsimony and explanatory power.

Fifth, focusing on entrepreneurial attitude as a dependent variable, rather than entrepreneurial intention or behavior, limits the ability to link findings to tangible behavioral outcomes. TPB posits that attitude influences intention, and intention predicts behavior, but these relationships are not deterministic. Nayak ^5^ reported that their model explains R ^2^ = .81 for intention but only R ^2^ = .59 for behavior, showing that the intention-behavior gap remains substantial. Future research should extend the model by incorporating entrepreneurial intention as an additional mediator between attitude and behavior, and collect objective behavioral indicators—registration in entrepreneurial skills, business plan development, legal incorporation of companies—in longitudinal follow-ups. This would make it possible to assess whether the suggested interventions (development of creativity and family involvement) effectively translate into consolidated ventures that contribute to reducing the devastating 90% failure rate in the first year in Peru.

### 4.4 Directions for future research

Four lines of research emerge as priorities for advancing knowledge about the psychosocial determinants of university entrepreneurship in emerging economies. First, longitudinal designs with multiple measurements that allow for the estimation of cross-lagged panel models and the specification of causal directionality between motivation, creativity, family support, and attitude. An optimal design would include: (a) measurement T _0_ at university entry (baseline); (b) randomized experimental intervention with a control group and treatment groups receiving (i) creativity training, (ii) a family involvement program, or (iii) both combined; (c) measurement T _1_ immediately post-intervention (3 months); (d) follow-up measurement T _2_ (12 months); (e) measurement T _3_ of objective entrepreneurial behavior (24 months). This design would allow for estimating causal effects of interventions, identifying longitudinal development trajectories, and definitively linking psychosocial determinants to consolidated entrepreneurial behaviors.

Second, multilevel comparative research examining how institutional and ecosystemic characteristics moderate the identified psychosocial relationships. Specifically, studies that include: (a) multiple universities with systematic variation in entrepreneurial resources—presence/absence of incubators, investment in innovation infrastructure, mandatory entrepreneurship courses—allowing for the estimation of institution × psychosocial mechanism cross-effects; (b) multiple regions of Peru with heterogeneous entrepreneurial ecosystems—dynamic north versus underdeveloped southern highlands—identifying contextual specificities; (c) international comparisons with other Latin American countries of medium development (Colombia, Ecuador, Chile) to assess cross-cultural generalization within the region. These multilevel studies would specify whether creativity development and family involvement interventions operate uniformly or require specific contextual adaptations.

Third, research on additional moderators that refine the understanding of the model’s boundary conditions. Candidate variables include: (a) gender, given that ^14^ demonstrated with 466 Vietnamese female students that family support counteracts perceptions of gender inequality; (b) family socioeconomic status, following evidence from ^12^ on economic status moderation; (c) family business background, distinguishing between generic family support versus specific entrepreneurial mentoring; (d) academic discipline, contrasting students in technical fields (engineering, sciences) versus social fields (business, economics) versus creative fields (design, architecture), given that the nature of creativity required may vary; (e) exposure to entrepreneurial role models, assessing whether the presence of successful entrepreneurs in personal networks amplifies or replaces the effect of family support. These multiple moderation analyses would allow for the design of differentiated and targeted interventions for specific subgroups.

Fourth, studies that integrate objective behavioral measures and methodological triangulation to overcome self-reporting limitations. This would include: (a) blind assessment of creativity through analysis of prototypes, business models, or innovative solutions by trained external judges, using standardized rubrics of originality, feasibility, and social impact; (b) observable behavioral indicators of entrepreneurial behavior—registered participation in competitions, presentation of projects to investors, legal incorporation of startups in SUNAT, attraction of external financing—; (c) objective metrics of family support—documented financial amounts, time invested by parents in the student’s entrepreneurial activities, verified introduction to family business networks; (d) diary studies in which students record their creative activities, interactions with family about entrepreneurship, and evolution of attitudes on a daily basis over extended periods, capturing intra-individual temporal dynamics. This methodological triangulation would substantially strengthen construct validity and allow psychosocial determinants to be linked to tangible entrepreneurial outcomes that contribute to the Sustainable Development Goals, particularly SDG 8 on decent work and economic growth in emerging economies with high youth unemployment.

## 5. Conclusions

The findings from this study of 600 first-year university students in northern Peru establish that entrepreneurial creativity fully mediates the relationship between intrinsic motivation and entrepreneurial attitude. The indirect effect (β = .25, 95% CI [.19, .32]) was significant, whereas the direct effect was not (β = .05, p = .214), demonstrating that intrinsic motivation operates exclusively through the development of creative capacities to shape entrepreneurial attitudes in this population.

Perceived family support functions as a significant moderator of the creativity-attitude pathway (β = .11, p < .001). The effect of creativity on attitude increases from β = .39 at low family support to β = .65 at high family support, with a critical threshold identified at 2.3 on the 1–5 scale through Johnson-Neyman analysis.

The integrated model explains 51% of the variance in entrepreneurial attitude (R ^2^ = .51), with superior predictive validity compared to the base model (Q ^2^ = .32 vs .26; MAE = .46 vs .52; RMSE = .58 vs.63).

Intrinsic motivation strongly predicts creativity (β = .48, f ^2^ = .30), while extrinsic motivation shows a small but significant effect (β = .12, f ^2^ = .02). Neither type of motivation demonstrates significant direct effects on entrepreneurial attitude.

Measurement invariance analyses confirmed that the instrument operates equivalently across public and private universities (ΔCFI ≤ .010, ΔRMSEA ≤ .015), and multigroup analyses revealed no significant structural differences between institution types.

These empirical results demonstrate that, in collectivist contexts such as Peru, the translation of motivational potential into entrepreneurial attitudes among first-year university students requires both the systematic development of creative capacities and the presence of family support structures that amplify this developmental process.

### Institutional review board statement

The study received ethical approval from the Research Ethics Committee of the School of Systems Engineering at César Vallejo University (Report No. 00429-2025/CEI-EIS, July 29, 2025).

### Informed consent statement

Written informed consent in electronic format was obtained from all participants via a mandatory confirmation checkbox in Google Forms prior to questionnaire access.

## Data Availability

All materials required for full reproducibility–the 600 anonymized individual responses, the variable codebook, the full 31‐item CDPAE instrument with construct‐dimension mapping, the descriptives and correlation matrix, the CFA standardized loadings, the PROCESS Model 14 path coefficients with bootstrap 95% CIs, and the configural‐metric‐scalar invariance tests by university type and gender–are openly deposited in the project's Figshare record (DOI: 10.6084/m9.figshare.31359715) under a CC-BY 4.0 licence, and are referenced below. Figshare: Dataset: Cultivating Sustainable Entrepreneurial Ecosystems – How Creativity Transforms Motivation into Entrepreneurial Attitudes Among First-Year University Students. https://doi.org/10.6084/m9.figshare.31359715. ^59^ This project contains the following underlying data:
•
**
Underlying_Data_CDPAE.xlsx** (anonymized individual responses from 600 participants, computed construct scores, and variable codebook) Figshare: Dataset: Cultivating Sustainable Entrepreneurial Ecosystems – How Creativity Transforms Motivation into Entrepreneurial Attitudes Among First-Year University Students. https://doi.org/10.6084/m9.figshare.31359715. ^59^ This project contains the following extended data:
•
**
Extended_Data_CDPAE.xlsx** (descriptive statistics, correlation matrix, CFA factor loadings, PROCESS Model 14 path coefficients, bootstrap indirect effects, and measurement invariance tests) •
**CDPAE_Questionnaire.xlsx** (complete questionnaire with 31 items, construct mapping, dimensions, response scales, and original sources) Data are available under the terms of the Creative Commons Attribution 4.0 International license (CC-BY 4.0).
