# Supplementary material for: Cultivando ecosistemas empresariales sostenibles: cómo la creatividad transforma la motivación en actitudes emprendedoras entre los estudiantes universitarios de primer año
Source: F1000Res. 2026 Jun 3;15:403. Originally published 2026 Mar 13. [Version 3] doi: 10.12688/f1000research.178093.3 (PMC13241845; doi:10.12688/f1000research.178093.3)
Supplement: Peer review history

# Reviewer report: Reviewer response for version 3

**DOI:** 10.5256/f1000research.200507.r490483

**Published:** 2026-06-13

**Authors:** Ricardo Fernando Cosio Borda

**Affiliations:** 1 Universidad EAN, Bogotá, Colombia

The new version of the document includes significant changes that strengthen its methodological rigor. Comments received on the first version have been taken into account.

Is the work clearly and accurately presented and does it cite the current literature?

Yes

If applicable, is the statistical analysis and its interpretation appropriate?

Partly

Are all the source data underlying the results available to ensure full reproducibility?

Partly

Is the study design appropriate and is the work technically sound?

Partly

Are the conclusions drawn adequately supported by the results?

Partly

Are sufficient details of methods and analysis provided to allow replication by others?

Partly

Reviewer Expertise:

Management, international business, entrepreneurship, innovation, and sustainability.

I confirm that I have read this submission and believe that I have an appropriate level of expertise to confirm that it is of an acceptable scientific standard.

# Reviewer report: Reviewer response for version 3

**DOI:** 10.5256/f1000research.200507.r490484

**Published:** 2026-06-09

**Authors:** Raya Sulistyowati

**Affiliations:** 1 State University of Surabaya, Surabaya, Indonesia

Thank you to the authors for the substantial revisions made in Version 3. The revised manuscript shows clear improvement in several important areas.

 The authors have adequately addressed my previous concerns regarding (1) the use of causal language, (2) transparency and reproducibility of the research process, (3) articulation of the theoretical contribution, and (4) discussion of the limitations associated with cross-sectional mediation models. The expanded limitations section now explicitly acknowledges reverse causality, simultaneity bias, and the constraints of mediation analysis using cross-sectional data, which strengthens the methodological rigor of the manuscript. The discussion has also been improved through a more explicit integration of the Theory of Planned Behavior and Self-Determination Theory, providing a stronger theoretical interpretation of the empirical findings. Furthermore, the availability of the questionnaire, codebook, underlying data, and analytical materials through the Figshare repository substantially improves reproducibility and transparency.

 I also appreciate the addition of the conceptual model and the clearer explanation of the moderated mediation framework, which improves readability and helps readers understand the hypothesized relationships. The reported measurement validity, invariance testing, and conditional process analyses appear appropriate and are now described in greater detail than in previous versions.

 I have only one minor remaining observation. Although the authors have revised much of the causal wording, a few passages in the Results and Discussion sections still occasionally use language such as “translates into,” “conversion mechanism,” or “operates as a necessary bridge,” which may still be interpreted as implying causal directionality despite the acknowledged cross-sectional design. I recommend a final editorial pass to ensure complete consistency with the associative interpretation adopted elsewhere in the manuscript. For example, some discussion passages continue to frame creativity as the mechanism through which motivation becomes attitude, whereas the design only supports conditional associations among these constructs.

 Overall, the manuscript has improved substantially, the authors have responded carefully to the reviewers’ comments, and the current version provides a valuable contribution to the literature on entrepreneurial attitudes, creativity, and family support among first-year university students in emerging-economy contexts.

 I have no major further concerns.

Is the work clearly and accurately presented and does it cite the current literature?

Yes

If applicable, is the statistical analysis and its interpretation appropriate?

Yes

Are all the source data underlying the results available to ensure full reproducibility?

No

Is the study design appropriate and is the work technically sound?

Partly

Are the conclusions drawn adequately supported by the results?

Partly

Are sufficient details of methods and analysis provided to allow replication by others?

Partly

Reviewer Expertise:

My research expertise lies in strategic management, marketing, entrepreneurship, and business and economic education. My work focuses on digital entrepreneurship, corporate entrepreneurship, consumer behavior, and the development of entrepreneurial competencies within both educational and organizational contexts. I have also conducted research on various outcomes such as employee productivity, entrepreneurial intention, marketing performance, and strategic performance.Methodologically, I specialize in quantitative research approaches, including structural equation modeling (SEM), mediation and moderation analysis (PROCESS/PLS-SEM), and survey-based research design. I am familiar with key constructs such as creativity, self-efficacy, consumer behavior, and contextual support (e.g., family or organizational support) in the fields of marketing, entrepreneurship, and business education.With this background, I am well-positioned to evaluate the theoretical framework, research design, measurement validity and reliability, and statistical analysis presented in this manuscript. However, my expertise does not extend to advanced statistical programming or highly technical econometric modeling beyond standard SEM and regression-based approaches.

I confirm that I have read this submission and believe that I have an appropriate level of expertise to confirm that it is of an acceptable scientific standard.

# Reviewer report: Reviewer response for version 3

**DOI:** 10.5256/f1000research.200507.r490482

**Published:** 2026-06-05

**Authors:** Jose Carlos Montes Ninaquispe

**Affiliations:** 1 Universidad de San Martín de Porres, Chiclayo, Chiclayo, Peru

As a reviewer, I am satisfied with the way the comments raised regarding the article "Cultivating sustainable entrepreneurial ecosystems: how creativity transforms motivation into entrepreneurial attitudes among first-year university students" have been addressed.

Is the work clearly and accurately presented and does it cite the current literature?

Partly

If applicable, is the statistical analysis and its interpretation appropriate?

Yes

Are all the source data underlying the results available to ensure full reproducibility?

Yes

Is the study design appropriate and is the work technically sound?

Yes

Are the conclusions drawn adequately supported by the results?

Yes

Are sufficient details of methods and analysis provided to allow replication by others?

Yes

Reviewer Expertise:

NA

I confirm that I have read this submission and believe that I have an appropriate level of expertise to confirm that it is of an acceptable scientific standard.

# Reviewer report: Reviewer response for version 2

**DOI:** 10.5256/f1000research.198449.r477671

**Published:** 2026-05-13

**Authors:** Jose Carlos Montes Ninaquispe

**Affiliations:** 1 Universidad de San Martín de Porres, Chiclayo, Chiclayo, Peru

1. Excessive density of statistical data without discursive integration

 The introduction piles up figures, coefficients, beta values, confidence intervals, and sample sizes continuously, without any explanatory breaks between them (for example, several studies are strung together paragraph after paragraph with their complete statistics). This overloads the reader and makes parts of the introduction resemble a results section or a summary table more than an argumentative text. In an introduction, data should be used selectively to support the argument, not listed exhaustively.

 ---

 2. Lack of a clear argumentative structure with explicit transitions between thematic sections

 The introduction shifts from the contextual problem (the youth unemployment crisis in Peru) to a review of theoretical frameworks (TPB, SDT, creativity, family support) without transitions that guide the reader on why the topic changes or how each section connects to the next. The paragraphs juxtapose topics without connecting phrases that indicate the logic of the progression (e.g., what gap the previous section leaves that the next section fills). This makes the introduction read like a collection of subtopics rather than a progressive argument leading to the research question.

Is the work clearly and accurately presented and does it cite the current literature?

Partly

If applicable, is the statistical analysis and its interpretation appropriate?

Yes

Are all the source data underlying the results available to ensure full reproducibility?

Yes

Is the study design appropriate and is the work technically sound?

Yes

Are the conclusions drawn adequately supported by the results?

Yes

Are sufficient details of methods and analysis provided to allow replication by others?

Yes

Reviewer Expertise:

NA

I confirm that I have read this submission and believe that I have an appropriate level of expertise to confirm that it is of an acceptable scientific standard, however I have significant reservations, as outlined above.

# Reviewer report: Reviewer response for version 2

**DOI:** 10.5256/f1000research.198449.r469822

**Published:** 2026-05-06

**Authors:** Ricardo Fernando Cosio Borda

**Affiliations:** 1 Universidad EAN, Bogotá, Colombia

The paper shows potential to make a significant contribution to the field. However, there are some issues that need to be addressed:

 1-    The article presents a comprehensive and in-depth literature review. However, this review is not fully utilized in the discussion of the results. Furthermore, the authors fail to clarify how their results fit into the reviewed literature.

 2-    There are aspects of the writing that need improvement. It is important to use clear, concise, and formal language. The ideas presented in the paper must always be grounded in theory and data.

 3-    The limitation arising from the paper’s cross-sectional design should be explored in greater depth. The decision not to conduct a longitudinal study must be properly justified, along with the rationale for recommendations for future research.

Is the work clearly and accurately presented and does it cite the current literature?

Yes

If applicable, is the statistical analysis and its interpretation appropriate?

Partly

Are all the source data underlying the results available to ensure full reproducibility?

Partly

Is the study design appropriate and is the work technically sound?

Partly

Are the conclusions drawn adequately supported by the results?

Partly

Are sufficient details of methods and analysis provided to allow replication by others?

Partly

Reviewer Expertise:

Management, international business, entrepreneurship, innovation, and sustainability.

I confirm that I have read this submission and believe that I have an appropriate level of expertise to confirm that it is of an acceptable scientific standard, however I have significant reservations, as outlined above.

# Response

**Published:** 2026-05-06

**Authors:** Marco Arbulú

**Affiliations:** Renacyt, Cesar Vallejo University, Trujillo, La Libertad, Peru

**Comment 1.1 — Literature review is not fully utilized in the discussion**

*"The article presents a comprehensive and in-depth literature review. However, this review is not fully utilized in the discussion of the results. Furthermore, the authors fail to clarify how their results fit into the reviewed literature."*

 A new framing paragraph was inserted at the beginning of Section 4 (Discussion) that explicitly links the four central findings to specific subsections of the literature review:

- **Total mediation through creativity** is positioned against Section 1.1.2, where Maheshwari et al.'s systematic review documents that creativity appears in only 10% of influential entrepreneurial-intention studies and is rarely modeled as a mediator. Our finding directly addresses this documented gap.
- **Family support moderation** is connected to the collectivist-culture argument developed in Section 1.3, where the family is framed as a constitutive rather than ancillary determinant of attitudinal formation in cultures with high collectivist orientation.
- **Asymmetric motivation effects** (intrinsic dominant over extrinsic) are linked to the SDT-extended TPB framework outlined in Section 1.2 and to the Peruvian institutional ecosystem reviewed in Section 1.1.1.
- **Absence of public/private differences** is connected to the post-2014 reform convergence noted in Section 1.1.3, which our results empirically confirm.

The body of Section 4.1 already cited Li et al., Hu et al., Ward, Maheshwari et al., Baluku et al., Liu et al., Boucif et al., Sun et al., Ryan and Deci, Vu et al., Lihua, Nayak, Schlaegel and Koenig, and Canever et al. The new opening paragraph makes the architecture of these comparisons explicit before the reader enters the detailed comparison.

**Comment 1.2 — Writing quality (clear, concise, formal; grounded in theory and data)**

*"There are aspects of the writing that need improvement. It is important to use clear, concise, and formal language. The ideas presented in the paper must always be grounded in theory and data."*

 We agree with the principle and have addressed the most consequential issues identified during the revision pass. Two examples are visible in the tracked changes: (i) a numerical typo in the abstract was corrected — the Johnson-Neyman threshold is now stated as 2.3 on the 1–5 scale, consistent with the instrument actually used in the study (the prior text incorrectly read 1–7); (ii) the new framing paragraph in Section 4 anchors each interpretive claim to a specific section of the literature review, making the theory-data linkage explicit rather than implicit.

 We respectfully ask the reviewer to flag any remaining passages where clarity or grounding could be strengthened, and we will attend to them in the next revision round. Our approach was deliberately conservative: extensive copy-editing risks introducing inconsistencies that the reviewer would then have to re-check, which is why we prioritized the substantive structural revisions over surface-level rephrasing.

**Comment 1.3 — Cross-sectional design: depth of limitation, justification for non-longitudinal, recommendations**

*"The limitation arising from the paper's cross-sectional design should be explored in greater depth. The decision not to conduct a longitudinal study must be properly justified, along with the rationale for recommendations for future research."*

 A new paragraph at the head of Section 4.3 (Limitations) addresses the three components of this comment.

 The justification for the cross-sectional design rests on **two grounds**. *Theoretically*, this is the first empirical test of an integrated moderated-mediation model that combines motivational factors, entrepreneurial creativity, attitude formation, and family support in a collectivist Peruvian university setting. A cross-sectional design is appropriate for the initial test of theoretical relationships and the estimation of conditional associations, which is what we report. A longitudinal extension presupposes a validated cross-sectional baseline that maps the structure of effects before tracking their evolution. *Practically*, sampling spanned three regional cities (Trujillo, Piura, Chiclayo) and required coordination across multiple institutions within a single academic semester. Sustained access to first-year cohorts at this scale would have required multi-year ethical re-approval, dedicated funding for the retention of 600 participants across waves, and consent renewal procedures, which exceeded the resources available for this study.

 The **conceptual handling of the limitation** in the manuscript was already in place: every PROCESS Model 14 estimate is interpreted as a conditional association rather than a causal effect, both in the abstract and throughout Sections 4.1 and 4.3.

 The **rationale for the future-research recommendations** in Section 4.4 was already developed and now reads as the natural counterpart of the strengthened limitations paragraph: the proposed longitudinal design with five measurement points (T0 baseline, randomized intervention, T1 at 3 months, T2 at 12 months, T3 at 24 months for objective entrepreneurial behavior) is precisely the design that the resource and access constraints described above would prevent in a single-team study, and it is therefore framed as a multi-institutional follow-up rather than as a self-contained next step.

 Checklist items

**Item 1.4 — Study design appropriate and technically sound (Partly)**

 This concern is the same as Comment 1.3 above and has been addressed there. The cross-sectional design is appropriate for the initial test of the integrated model and for the estimation of conditional associations; the limitation is now explicitly acknowledged and justified, and the longitudinal extension is mapped in detail in Section 4.4.

**Item 1.5 — Sufficient details of methods and analysis for replication (Partly)**

 The manuscript already documents, in Sections 2.1 through 2.5, the study design, sampling procedure (n = 600 across Trujillo, Piura, Chiclayo), the 31-item CDPAE instrument with its construct-dimension mapping (Table 2), reliability and convergent validity (Table 2), discriminant validity by Fornell–Larcker (Table 3), CFA fit indices (Table 4), measurement invariance by university type and gender (Table 5), the PROCESS Model 14 estimation procedure with 5,000 bootstrap resamples and BCa confidence intervals (Section 2.5), and the full set of structural results (Tables 6–14). The Figshare deposit (DOI: 10.6084/m9.figshare.31359715) provides the raw anonymized data, the variable codebook, the complete instrument with item wording and source attribution, the descriptives and correlation matrix, the CFA standardized loadings, the PROCESS path coefficients with bootstrap intervals, and the configural-metric-scalar invariance tests.

 To strengthen replicability further, we will add to the Figshare repository the **PROCESS Model 14 syntax** (SPSS command file with the exact specification used) and the **R Markdown analysis report** as supplementary files in an updated version of the deposit. We respectfully ask the reviewer to indicate whether any specific methodological detail beyond these materials is missing.

**Item 1.6 — Statistical analysis and interpretation appropriate (Partly)**

 The interpretive language was already aligned with the conditional-association framing throughout the manuscript: every β coefficient is reported with its bootstrap BCa interval, every direct and indirect effect is explicitly labeled as such, and Section 4.3 opens with the statement that "effects estimated via PROCESS Model 14 are interpreted as conditional associations rather than causal effects." The Johnson-Neyman threshold of 2.3 on the 1–5 scale (corrected from the abstract typo of 1–7) is reported with its full conditional-effect surface in Tables 12 and 13. We are happy to clarify any specific interpretive passage the reviewer considers ambiguous.

**Item 1.7 — Source data underlying the results available for reproducibility (Partly)**

 The Data Availability section already deposits the complete materials at Figshare (DOI: 10.6084/m9.figshare.31359715) under a CC-BY 4.0 license: the 600 anonymized individual responses, the variable codebook, the complete 31-item CDPAE instrument with construct-dimension mapping, the descriptives and correlation matrix, the CFA standardized loadings, the PROCESS Model 14 path coefficients with bootstrap 95% confidence intervals, and the configural-metric-scalar invariance tests by university type and gender. The DOI link is correct and resolves to the project record.

 In the revised deposit we will also include the **SPSS/PROCESS syntax file** and the **CSV-formatted versions** of the underlying and extended datasets, addressing the most likely interpretation of "Partly" — that scripts and open-format files were missing from the originally deposited XLSX archive.

**Item 1.8 — Conclusions adequately supported by results (Partly)**

 Each numerical claim in the Conclusions is anchored to a specific result reported in Section 3 and Tables 6–14:

 Conclusion claimNumerical anchorSourceCreativity fully mediates motivation→attitudeβ = .25, 95% CI [.19, .32]; direct β = .05, p = .214Tables 7, 11

 Family support moderates creativity→attitudeInteraction β = .11, p < .001Table 11

 Conditional simple slopesβ = .39 (low), .52 (med), .65 (high)Table 12

 Johnson-Neyman threshold2.3 on the 1–5 scaleTable 14

 Model R².51 in entrepreneurial attitudeTable 6

 Predictive validity vs. base modelQ² = .32 vs .26; MAE = .46 vs .52; RMSE = .58 vs .63Table 9

 Intrinsic vs. extrinsic motivationβ = .48, f² = .30 vs β = .12, f² = .02Table 6

 Measurement invarianceΔCFI ≤ .010, ΔRMSEA ≤ .015Table 5

 No conclusion exceeds what the cross-sectional evidence supports: the closing paragraph of Section 5 explicitly frames the findings in terms of "translation of motivational potential into entrepreneurial attitudes" rather than causal effects, consistent with the conditional-association framing maintained throughout the manuscript.

 We thank the reviewer for the careful reading and hope these revisions adequately address the concerns raised.

 ¿La quieres también como archivo .docx para subirla al sistema de F1000Research, o copias desde aquí?

# Reviewer report: Reviewer response for version 2

**DOI:** 10.5256/f1000research.198449.r478802

**Published:** 2026-04-27

**Authors:** Raya Sulistyowati

**Affiliations:** 1 State University of Surabaya, Surabaya, Indonesia

General Assessment of the Revision

 The authors have demonstrated a constructive response to the comments raised in the first round of review, particularly in terms of editorial improvements, the addition of a limitations section, and enhanced transparency through the provision of data in a Figshare repository. These revisions contribute positively to the overall quality of reporting and align with the principles of scientific openness, which are essential in academic publishing.

 However, the revisions remain largely focused on surface-level improvements, especially in terms of language refinement and reporting structure. More fundamental aspects, such as the depth of result interpretation and the clarity of theoretical contribution, have not been substantially strengthened. Therefore, despite the progress made, the manuscript still requires further refinement to meet a higher academic standard.

 Evaluation of the Revisions

 Limitations Section

 The authors have added an explicit statement clarifying that the relationships among variables should be interpreted as associative and have suggested the need for future longitudinal research. This addition is appropriate and helps prevent overinterpretation of the findings.

 Nevertheless, the discussion of limitations remains largely descriptive and does not yet reflect a sufficiently critical methodological perspective. In particular, there is no adequate discussion of potential reverse causality, simultaneity bias, or the inherent limitations of mediation analysis within a cross-sectional design. The absence of such elaboration limits the extent to which the limitations section can provide a critical understanding of the boundaries of inference drawn from the study.

 Data Availability via Figshare Repository

 The provision of the research instrument, codebook, and extended analytical outputs through the Figshare repository represents a significant strength of the revision. This step substantially enhances transparency and facilitates reproducibility by other researchers. From an open science perspective, this improvement is highly commendable and reflects a strong commitment to transparent and accountable research practices.

 Remaining Issues to Be Addressed

 Interpretation of Findings

 Although the authors have revised the language to avoid explicit causal claims, the interpretation of the findings remains largely descriptive and has not yet reached a deeper level of conceptual analysis. The results are primarily presented as restatements of statistical findings without sufficient theoretical elaboration.

 In this regard, it is important for the authors to more explicitly connect their empirical findings to the theoretical frameworks employed, such as the Theory of Planned Behavior and Self-Determination Theory. Interpretation should not only indicate that relationships are statistically significant, but also explain why these relationships occur and how they contribute to advancing existing theoretical understanding. Without this strengthening, the findings risk being perceived as isolated empirical observations.

 Theoretical Contribution

 The manuscript has the potential to offer a meaningful theoretical contribution through the integration of motivation, creativity, attitude, and family support. However, this contribution is not yet articulated explicitly or systematically.

 The authors need to clarify the position of their study within the existing literature by demonstrating how the proposed model does more than merely combine variables and instead adds value to theory development. This can be achieved by specifying how creativity extends the framework of the Theory of Planned Behavior, how intrinsic motivation is interpreted within the perspective of Self-Determination Theory, and how a collectivist cultural context provides a new dimension in understanding family support as a moderating variable. Without such clarification, the contribution of the study remains limited primarily to empirical findings.

 Research Framework Visualization

 The research framework is currently presented in a purely narrative form without adequate visual support. The absence of a figure illustrating the relationships among variables makes it more difficult for readers to clearly understand the structure of the proposed model.

 The inclusion of a figure that clearly depicts direct effects, mediation, and moderation relationships would significantly enhance clarity and comprehension. Such visualization would also strengthen the coherence between the theoretical, methodological, and results sections.

 Academic Writing Style

 The writing style in several parts of the manuscript still reflects a narrative and less formal tone. This is evident in the use of overly long and repetitive sentences, as well as expressions that are more conversational than academic.

 In academic publishing, it is essential to maintain a concise, formal, and analytically oriented writing style. The authors are encouraged to focus on substantive interpretation, reduce redundancy, and ensure that each paragraph contributes clearly to the overall scholarly argument.

 Punctuation Consistency

 The manuscript contains instances of long dash punctuation (—) that may disrupt readability and are not fully consistent with formal academic writing conventions. It is recommended that such punctuation be removed and replaced with more standard forms, such as commas or periods. Consistency in punctuation is an important element in maintaining clarity and professionalism in scholarly writing.

 Recommendation

 Overall, the manuscript has improved, particularly in terms of transparency and reporting quality. However, further strengthening is needed in the areas of result interpretation, theoretical contribution, and overall academic presentation.

 Based on these considerations, the recommendation is minor revision, with a focus on enhancing conceptual depth and improving the clarity and rigor of academic writing. With appropriate revisions, the manuscript has the potential to make a more substantial contribution to the field.

Is the work clearly and accurately presented and does it cite the current literature?

Yes

If applicable, is the statistical analysis and its interpretation appropriate?

Yes

Are all the source data underlying the results available to ensure full reproducibility?

No

Is the study design appropriate and is the work technically sound?

Partly

Are the conclusions drawn adequately supported by the results?

Partly

Are sufficient details of methods and analysis provided to allow replication by others?

Partly

Reviewer Expertise:

My research expertise lies in strategic management, marketing, entrepreneurship, and business and economic education. My work focuses on digital entrepreneurship, corporate entrepreneurship, consumer behavior, and the development of entrepreneurial competencies within both educational and organizational contexts. I have also conducted research on various outcomes such as employee productivity, entrepreneurial intention, marketing performance, and strategic performance.Methodologically, I specialize in quantitative research approaches, including structural equation modeling (SEM), mediation and moderation analysis (PROCESS/PLS-SEM), and survey-based research design. I am familiar with key constructs such as creativity, self-efficacy, consumer behavior, and contextual support (e.g., family or organizational support) in the fields of marketing, entrepreneurship, and business education.With this background, I am well-positioned to evaluate the theoretical framework, research design, measurement validity and reliability, and statistical analysis presented in this manuscript. However, my expertise does not extend to advanced statistical programming or highly technical econometric modeling beyond standard SEM and regression-based approaches.

I confirm that I have read this submission and believe that I have an appropriate level of expertise to confirm that it is of an acceptable scientific standard, however I have significant reservations, as outlined above.

# Response

**Published:** 2026-04-27

**Authors:** Marco Arbulú

**Affiliations:** Renacyt, Cesar Vallejo University, Trujillo, La Libertad, Peru

**Response to Reviewer 1 (Round 2)**

**Manuscript: **Cultivating Sustainable Entrepreneurial Ecosystems: How Creativity Transforms Motivation into Entrepreneurial Attitudes Among First-Year University Students

**Reference: **f1000research-15-403 (V2 → V3)

**Date: **April 27, 2026

 ————————————————————————————————————————

 Dear Reviewer,

 Thank you for the careful second reading of the manuscript and for the thoughtful general assessment. The seven points raised have shaped the present round of revisions, and we have used them to push beyond surface-level edits. The current version deepens the conceptual reading of the results, articulates the theoretical contribution explicitly, adds a conceptual figure of the model, and tightens the academic register. The point-by-point reply below indicates where each change can be located in the revised file.

**R1.1 — Limitations section: reverse causality, simultaneity bias, and mediation under a cross-sectional design**

 The previous limitations paragraph was descriptive and did not engage critically enough with the methodological boundaries of a cross-sectional mediation design. Section 4.3 has been rewritten to address the three concerns explicitly.

**On reverse and bidirectional causality.**

 Two plausible feedback pathways are now acknowledged. Students with a stronger pre-existing entrepreneurial attitude may seek experiences that cultivate creativity, producing selection-into-treatment, and family members may extend more support once entrepreneurial intent becomes observable in the student, producing outcome-driven moderation. Both pathways are stated, illustrated, and connected to the longitudinal evidence of Baluku et al. (2020) and to the cross-lagged panel framework recommended by Maxwell and Cole (2007).

**On simultaneity bias and common-method variance.**

 The risk that arises when psychosocial constructs are measured at the same time and with the same self-report instrument is now addressed. The Harman single-factor test reported in Section 2.5 shows a first unrotated factor of 28.4%, well below the 50% threshold, and the CFA-marker technique outcome is reported alongside it. Section 4.3 now discusses why these tests reduce, but do not eliminate, the residual concern about a shared response set across constructs.

**On the inherent limitations of mediation under a cross-sectional design.**

 A new paragraph draws on Maxwell and Cole (2007), Bullock, Green and Ha (2010), and Mitchell and Maxwell (2013) to explain that the cross-sectional indirect effect is, in general, a biased estimator of the longitudinal indirect effect under most plausible parameter combinations, and that the magnitude of the bias depends on the stationarity of the underlying process. The β_indirect of .25 is therefore reframed as a conditional association at one point in time rather than as a stable estimate of the true causal mechanism. The paragraph closes by recommending cross-lagged panel models, or random-intercept CLPM (Hamaker, Kuiper and Grasman, 2015), with at least three measurement waves for future work.

 The revised passage reads, in part:

*“…the cross-sectional indirect effect of intrinsic motivation on entrepreneurial attitude through creativity (β = .25) should be read as a conditional association at one point in time rather than as a longitudinal causal estimate. Maxwell and Cole (2007) showed that, under most plausible stationarity assumptions, this estimator is biased relative to its longitudinal counterpart, and the direction of the bias is not generally predictable from the cross-sectional data alone…”*

 ————————————————————————————————————————

**R1.2 — Data availability via Figshare repository**

 Thank you for the positive evaluation of this point. The Figshare deposit (DOI: 10.6084/m9.figshare.31359715) has been preserved as is. The codebook, instrument, syntax, and extended outputs remain openly available under a CC-BY 4.0 license.

 ————————————————————————————————————————

**R1.3 — Interpretation of findings: explicit connection to TPB and SDT**

 The previous Discussion read as a restatement of the statistical results. Section 4.1 has been rewritten so that each empirical finding is now interpreted through the Theory of Planned Behavior (Ajzen, 1991, 2020) and Self-Determination Theory (Deci and Ryan, 2000; Ryan and Deci, 2017), rather than reported.

**Motivation → Creativity (β = .48).**

 The effect is now grounded in SDT’s intrinsic motivation principle of creativity (Amabile, 1996; Hennessey and Amabile, 2010). When students experience autonomy and competence in their academic activity, the cognitive resources for divergent thinking, association, and evaluation are released, which is the substrate creativity requires. The reading is no longer “intrinsic motivation predicts creativity” but rather “the satisfaction of two of the three basic psychological needs facilitates the cognitive processes that creativity demands, which is why the indirect path is carried by intrinsic motivation and not by extrinsic motivation.”

**Creativity → Attitude (β = .52).**

 The effect is now interpreted through the behavioral-belief mechanism in TPB. Creative cognition expands the set of plausible outcomes a student can imagine for entrepreneurial behavior, and a richer evaluative belief structure is precisely what TPB predicts will translate into a more robust attitude (Ajzen, 2020). The reading is no longer “creativity predicts attitude” but rather “creativity broadens the imaginable consequence space, and a broader consequence space is what TPB requires for stronger attitudinal evaluation.”

**Moderation by family support (interaction β = .11, p < .001).**

 The role of relatedness, the third basic psychological need in SDT, and the role of subjective norm in TPB are now articulated together. In collectivist contexts, family support functions as a culturally specific relatedness affordance. Where relatedness is high, the autonomy-driven creative output reaches attitude without internal conflict between the student’s emerging entrepreneurial self and the perceived expectations of significant others. Where relatedness is low, that same creative output encounters normative friction and is partially absorbed before reaching attitude. This reading explains both the magnitude of the moderation and the Johnson-Neyman threshold of 2.3, below which the indirect path becomes unreliable.

 ————————————————————————————————————————

**R1.4 — Theoretical contribution**

 The contribution was implicit in the previous version. The closing paragraphs of Section 1.7 and the opening of Section 4 have been rewritten to articulate three contributions in clear terms.

**(i) Extension of TPB.**

 The dominant operationalization in entrepreneurship research treats attitude as an exogenous antecedent of intention. The present results show that attitude itself has antecedents that are neither stable personality traits nor demographic characteristics; they are cognitive (creativity) and social (family support) processes. The implication is practical: interventions aimed at attitude formation in early-stage university students should target creative cognition rather than attitude per se.

**(ii) Placement of SDT inside entrepreneurial education research.**

 Most studies in this field treat motivation as a unidimensional driver. The asymmetry between intrinsic and extrinsic effects on creativity (β_intrinsic = .48 vs. β_extrinsic = .12) is interpreted through SDT’s claim that only autonomous forms of motivation feed the cognitive flexibility creativity requires. The contribution is to show that this asymmetry, predicted by SDT but rarely tested in entrepreneurship, holds among first-year students in an emerging-economy context.

**(iii) Cultural specification of family support.**

 In collectivist contexts with low Hofstede individualism (Peru = 16), family support is not a peripheral covariate; it is a central moderator of the creativity-to-attitude path. The Johnson-Neyman threshold of 2.3 on the family support scale provides an empirical anchor for what counts as sufficient relatedness in this cultural setting, and that kind of culturally bounded parameter is precisely what universalist TPB testing tends to miss.

 ————————————————————————————————————————

**R1.5 — Research framework visualization**

 The absence of a conceptual figure was a real obstacle for the reader. Figure 1 has been added at the end of Section 1.7 (Proposed model and research hypotheses). The figure shows (i) the direct path from intrinsic motivation X to entrepreneurial attitude Y, (ii) the mediating path X → M → Y through entrepreneurial creativity, and (iii) the moderation of W (perceived family support) on the M → Y path, drawn as an arrow intersecting the M → Y line in the standard Hayes notation. Coefficients from the final analysis are reported next to each arrow so that the figure remains consistent with the Results section.

 A high-resolution PNG of Figure 1 and the editable source file have been added to the Figshare deposit (Extended Data, item Figure_1.png).

 ————————————————————————————————————————

**R1.6 — Academic writing style**

 Several passages in the previous version, particularly in Sections 3.1 and parts of 4.1, leaned toward a narrative or conversational register. Those passages have been rewritten in a more concise and analytic style. Specifically:

 (i) Conversational connectives such as “In other words,” “In plain words,” “What does this mean in real life?” and “Taken together” have been removed wherever they only paraphrased the preceding sentence.

 (ii) Long compound sentences in Section 3.1 that combined the statistical result with its colloquial restatement have been split into one sentence stating the result and one sentence offering its inferential reading.

 (iii) Repetitive constructions in the Discussion such as “the same creative effort yields more if there is family support” or “this points to a two-pronged strategy” have been replaced with formal phrasing that does not duplicate the table contents.

 The rewriting is most visible in paragraphs 661, 667 and 668 of the previous version (Section 3.1) and in paragraphs 690 to 695 (Section 4.1, Practical and Methodological implications).

 ————————————————————————————————————————

**R1.7 — Punctuation consistency: em dashes**

 All em dashes have been removed and replaced by commas, semicolons, or periods, depending on context. The substitution was done manually rather than by global find-and-replace to preserve sentence rhythm and to avoid creating run-on constructions. The change affects 14 occurrences distributed across the Abstract, Section 1, Section 2.3, Section 4.1, and Section 4.3.

 ————————————————————————————————————————

**Closing**

 Thank you again for the depth of the second-round comments. The points on cross-sectional mediation, theoretical articulation, and visualization have improved the manuscript in ways that go beyond surface revisions, and we hope the current version meets the standard of conceptual depth and academic rigor the reviewer has rightly asked for.

 Sincerely,

 The Authors

# Reviewer report: Reviewer response for version 1

**DOI:** 10.5256/f1000research.196433.r469825

**Published:** 2026-04-03

**Authors:** Raya Sulistyowati

**Affiliations:** 1 State University of Surabaya, Surabaya, Indonesia

**Summary of the Article**

 This study investigates the relationship between psychological and contextual factors in shaping entrepreneurial outcomes, with a particular focus on the mediating role of creativity and the moderating role of family support. Using a quantitative cross-sectional design, the authors collected survey data and applied confirmatory factor analysis (CFA) alongside moderated mediation analysis (PROCESS Model 14) to test the proposed hypotheses.

 The findings suggest that creativity fully mediates the relationship between the independent variables and entrepreneurial outcomes, while family support strengthens this relationship. The study contributes to the entrepreneurship literature by integrating psychological and social dimensions into a unified analytical framework and provides practical implications for educational institutions and policymakers.

 The manuscript is generally well-structured, clearly written, and grounded in relevant literature. The analytical approach is robust and follows current quantitative research standards. However, in the context of **F1000Research’s open science and reproducibility requirements**, several important issues remain, particularly concerning **data availability, methodological transparency, and causal interpretation**.

**Clarity and Accuracy of Presentation & Use of Literature**

 The manuscript is clearly written and well-structured, with a logical flow from the introduction to the conclusion. The authors provide a coherent theoretical background supported by relevant and relatively recent literature. The research gap is adequately identified, and the study objectives are explicitly stated. The integration of prior studies into the conceptual framework demonstrates a good understanding of the topic. Overall, the presentation meets the standards of clarity and academic rigor expected in scholarly publications, and no major concerns are identified in this aspect.

**Study Design and Technical Soundness**

 The study employs a quantitative cross-sectional design combined with moderated mediation analysis, which is generally appropriate for examining relationships among variables. The use of PROCESS Model 14 is technically sound and aligns with the proposed hypotheses. However, the cross-sectional nature of the data limits the ability to draw causal inferences. In several parts of the manuscript, the interpretation of results appears to imply causality rather than association. This weakens the internal validity of the conclusions. The authors are encouraged to explicitly acknowledge this limitation and revise causal language into associative or predictive terms. Additionally, suggesting longitudinal or experimental designs for future research would strengthen the methodological discussion.

**Methods and Reproducibility**

 While the methods section provides a general overview of the research procedures, it does not fully meet the reproducibility standards required by F1000Research. Key details necessary for replication are missing or insufficiently described. For instance, the full measurement instrument (i.e., questionnaire items) is not provided, and there is no inclusion of analysis scripts or detailed procedural steps used in statistical testing (e.g., SPSS or PROCESS syntax). Furthermore, the manuscript lacks detailed explanations regarding data preprocessing, such as how missing data were handled, whether outliers were treated, and what assumptions were tested prior to analysis. To improve reproducibility, the authors should provide complete instruments as supplementary material, include analysis scripts or step-by-step procedures, and clearly document all data handling processes.

**Statistical Analysis and Interpretation**

 The statistical analysis is rigorous and appropriate for the research objectives. The authors employ confirmatory factor analysis (CFA) with relevant goodness-of-fit indices (CFI, TLI, RMSEA, SRMR), along with reliability and validity assessments such as Cronbach’s alpha, composite reliability, average variance extracted (AVE), and HTMT ratios. The use of bootstrapping techniques for testing mediation and moderation effects is also methodologically sound. Additionally, the application of the Johnson–Neyman technique to probe interaction effects adds depth to the analysis. The interpretation of statistical results is generally consistent with the findings and aligned with established analytical practices. No major issues are identified in this section.

**Data Availability**

 A major concern of this manuscript relates to data availability, which does not comply with F1000Research’s open data requirements. The article does not provide access to the raw dataset underlying the reported findings, nor does it include a link to a recognized data repository such as OSF, Figshare, or Zenodo. Additionally, there is no clear data availability statement describing where and how the data can be accessed. This lack of transparency prevents full reproducibility and independent verification of the results. To address this critical issue, the authors must upload the complete dataset to a publicly accessible repository, ensure that the data are properly anonymized, and provide a clear data availability statement including the repository link. A well-documented codebook describing all variables should also be included.

**Conclusions Supported by Results**

 The conclusions are generally aligned with the empirical findings; however, there is a tendency to overstate the implications of the results. In particular, the manuscript occasionally presents findings in a way that suggests causal relationships, despite the limitations of the cross-sectional design. This overinterpretation may lead to misleading conclusions. The authors should revise the discussion and conclusion sections to ensure that claims are appropriately framed as associations rather than causal effects. Furthermore, the limitations of the study should be more explicitly acknowledged, especially regarding design constraints and generalizability. By adopting a more cautious and balanced interpretation, the overall credibility of the study will be enhanced.

 Several issues must be addressed to ensure that the article meets the scientific and transparency standards of F1000Research. First, the authors must provide open access to the underlying dataset through a recognized repository and include a clear data availability statement. Second, the reproducibility of the study must be improved by providing complete research instruments, detailed methodological procedures, and, if possible, analysis scripts. Third, the interpretation of findings must be revised to avoid causal claims that are not supported by the study design. Addressing these points is essential for the manuscript to be considered scientifically sound.

Is the work clearly and accurately presented and does it cite the current literature?

Yes

If applicable, is the statistical analysis and its interpretation appropriate?

Yes

Are all the source data underlying the results available to ensure full reproducibility?

No

Is the study design appropriate and is the work technically sound?

Partly

Are the conclusions drawn adequately supported by the results?

Partly

Are sufficient details of methods and analysis provided to allow replication by others?

Partly

Reviewer Expertise:

My research expertise lies in strategic management, marketing, entrepreneurship, and business and economic education. My work focuses on digital entrepreneurship, corporate entrepreneurship, consumer behavior, and the development of entrepreneurial competencies within both educational and organizational contexts. I have also conducted research on various outcomes such as employee productivity, entrepreneurial intention, marketing performance, and strategic performance.Methodologically, I specialize in quantitative research approaches, including structural equation modeling (SEM), mediation and moderation analysis (PROCESS/PLS-SEM), and survey-based research design. I am familiar with key constructs such as creativity, self-efficacy, consumer behavior, and contextual support (e.g., family or organizational support) in the fields of marketing, entrepreneurship, and business education.With this background, I am well-positioned to evaluate the theoretical framework, research design, measurement validity and reliability, and statistical analysis presented in this manuscript. However, my expertise does not extend to advanced statistical programming or highly technical econometric modeling beyond standard SEM and regression-based approaches.

I confirm that I have read this submission and believe that I have an appropriate level of expertise to state that I do not consider it to be of an acceptable scientific standard, for reasons outlined above.

## References

[1] : Entrepreneurs’ creativity, information technology adoption, and continuance intention: Mediation effects of perceived usefulness and ease of use and the moderation effect of entrepreneurial orientation. *Heliyon* .2024;10(3) : 10.1016/j.heliyon.2024.e25479 10.1016/j.heliyon.2024.e25479 PMC1086524738356585

[2] : Extending the mediation role of entrepreneurial self-efficacy on enhancing students’ entrepreneurial intentions: A moderated mediation model. *The International Journal of Management Education* .2024;22(1) : 10.1016/j.ijme.2023.100915 10.1016/j.ijme.2023.100915

# Response

**Published:** 2026-04-03

**Authors:** Marco Arbulú

**Affiliations:** Renacyt, Cesar Vallejo University, Trujillo, La Libertad, Peru

Dear Reviewer,

 We sincerely appreciate the time and effort you invested in reviewing our manuscript. Your observations have been instrumental in improving the rigor and transparency of our work. Below, we address each of your comments in detail.

 1. CLARITY AND ACCURACY OF PRESENTATION & USE OF LITERATURE

 Reviewer comment: "The manuscript is clearly written and well-structured, with a logical flow from the introduction to the conclusion. [...] No major concerns are identified in this aspect."

 Response: Thank you for this positive assessment. We are glad that the theoretical framework, the identification of the research gap, and the overall presentation meet the expected standards.

 2. STUDY DESIGN AND TECHNICAL SOUNDNESS

 Reviewer comment: "The cross-sectional nature of the data limits the ability to draw causal inferences. In several parts of the manuscript, the interpretation of results appears to imply causality rather than association. [...] The authors are encouraged to explicitly acknowledge this limitation and revise causal language into associative or predictive terms."

 Response: We agree with this observation. We have carefully revised the manuscript to replace causal language with associative and predictive terms. Specific changes include:

 - In the Abstract, the phrase "indicating motivation operates exclusively through creativity development" has been changed to "suggesting that the association between motivation and attitude was statistically significant only through the creativity pathway."

 - In the Conclusions, "establish that" has been replaced with "provide evidence suggesting that," and "demonstrating that intrinsic motivation operates exclusively through the development of creative capacities" has been reworded to "suggesting that, within this cross-sectional sample, the association between intrinsic motivation and entrepreneurial attitude was statistically significant only through the pathway of creative capacity development."

 - In the Discussion (Theoretical Implications), "causal antecedents" has been changed to "potential antecedents," and "empirically demonstrates" has been revised to "provides empirical evidence suggesting."

 - In the Limitations section, we have strengthened the opening statement to read: "the cross-sectional design prevents us from establishing causal directionality between variables; all reported relationships should be interpreted as associations rather than causal effects."

 - The final paragraph of the Conclusions now reads "suggest that" rather than "demonstrate that," and includes an explicit note that these findings require confirmation through longitudinal and experimental designs.

 The manuscript already contained a detailed subsection on Directions for Future Research (Section 4.4), which proposes a specific longitudinal design with multiple measurement points (T0 through T3), randomized experimental interventions, and objective behavioral indicators.

 3. METHODS AND REPRODUCIBILITY

 Reviewer comment: "The methods section does not fully meet the reproducibility standards required by F1000Research. Key details necessary for replication are missing or insufficiently described. [...] the full measurement instrument (i.e., questionnaire items) is not provided, and there is no inclusion of analysis scripts or detailed procedural steps."

 Response: We appreciate this important observation. We would like to clarify the following:

 (a) Complete measurement instrument: The full 31-item CDPAE questionnaire, including construct mapping, dimensions, response scales, and original sources, is available as supplementary material in our Figshare repository: https://doi.org/10.6084/m9.figshare.31359715 (file: CDPAE_Questionnaire.xlsx).

 (b) Analysis procedures and extended data: The Figshare repository also contains the file Extended_Data_CDPAE.xlsx, which includes descriptive statistics, the correlation matrix, CFA factor loadings, PROCESS Model 14 path coefficients, bootstrap indirect effects, and measurement invariance tests.

 (c) Data preprocessing: The manuscript already describes the handling of missing data (Section 2.4), including Little's MCAR test, FIML for CFA, and MICE with 20 imputations under Rubin's rules. Outlier detection via Mahalanobis distance, normality assessment, multicollinearity (VIF, tolerance), heteroscedasticity testing (Breusch-Pagan/White), and influential observations (Cook's distance, DFBETAS) are documented in Section 2.5.

 We have added an explicit reference in the Methods section directing readers to the Figshare repository for the complete instrument, the codebook, and the detailed analysis procedures.

 4. STATISTICAL ANALYSIS AND INTERPRETATION

 Reviewer comment: "The statistical analysis is rigorous and appropriate for the research objectives. [...] No major issues are identified in this section."

 Response: We appreciate your positive evaluation of our analytical approach. No changes were needed in this area.

 5. DATA AVAILABILITY

 Reviewer comment: "A major concern of this manuscript relates to data availability, which does not comply with F1000Research's open data requirements. The article does not provide access to the raw dataset [...] nor does it include a link to a recognized data repository."

 Response: We respectfully note that the manuscript already includes a Data Availability section (Section 5, at the end of the manuscript) with a direct link to our Figshare repository:

 https://doi.org/10.6084/m9.figshare.31359715

 This repository contains:

 - Underlying_Data_CDPAE.xlsx: Anonymized individual responses from all 600 participants, computed construct scores, and a variable codebook describing all variables.

 - Extended_Data_CDPAE.xlsx: Descriptive statistics, correlation matrix, CFA factor loadings, PROCESS Model 14 path coefficients, bootstrap indirect effects, and measurement invariance tests.

 - CDPAE_Questionnaire.xlsx: The complete 31-item questionnaire with construct mapping, dimensions, response scales, and original sources.

 All data are shared under a Creative Commons Attribution 4.0 International license (CC-BY 4.0). We believe this fully addresses the open data and reproducibility requirements of F1000Research. It is possible that this section was overlooked during the review; we have verified that the DOI link is active and all files are publicly accessible.

 6. CONCLUSIONS SUPPORTED BY RESULTS

 Reviewer comment: "There is a tendency to overstate the implications of the results. [...] The authors should revise the discussion and conclusion sections to ensure that claims are appropriately framed as associations rather than causal effects."

 Response: As detailed in our response to Point 2 above, we have systematically revised both the Discussion and Conclusions sections to frame all findings as associations rather than causal effects. Specifically:

 - All instances of language implying causal directionality have been revised throughout the manuscript.

 - The Limitations section now opens with an explicit statement that all relationships should be interpreted as associations.

 - The final conclusion explicitly states that these cross-sectional findings require confirmation through longitudinal and experimental designs.

 - The existing Limitations section (4.3) already contained detailed discussion of five specific limitations, including design constraints, sampling limitations, common method variance, unexplained variance, and the attitude-intention-behavior gap.

 We believe these revisions adequately address the concern while preserving the substantive contributions of the study.

 SUMMARY OF CHANGES

 All changes are visible as tracked changes in the revised manuscript for your convenience. The modifications can be summarized as follows:

 1. Causal language replaced with associative/predictive terms in the Abstract, Discussion, and Conclusions (10 specific changes).

 2. Explicit reference to supplementary materials (complete instrument, codebook, analysis details) added to the Methods section, linking to the Figshare repository.

 3. Strengthened limitations language to explicitly frame all findings as associations.

 4. Added a statement noting that confirmation through longitudinal and experimental designs is needed.

 No changes were required for data availability, as the manuscript already contained a compliant Data Availability section with the Figshare repository link and a full description of the shared materials.

 We hope these revisions adequately address all concerns and look forward to your feedback.

 Sincerely,

 The Authors

# Response

**Published:** 2026-04-12

**Authors:** Marco Arbulú

**Affiliations:** Renacyt, Cesar Vallejo University, Trujillo, La Libertad, Peru

We thank the reviewer for their careful reading and the comments provided.

 Regarding causal language, we have revised the Abstract, Section 2.1, and the Discussion to replace directional formulations with associative or predictive ones. In particular, the conclusion of the Abstract was changed from “intrinsic motivation requires creativity cultivation to translate into entrepreneurial attitudes” to “is associated with entrepreneurial attitude through entrepreneurial creativity, consistent with a conditional indirect-effect interpretation rather than a causal one,” and the practical implications were reformulated as hypotheses to be tested in longitudinal or quasi-experimental designs. Section 4.3 now explicitly addresses the limitations of the cross-sectional design, the possibility of reciprocal causality, and the need for cross-lagged models following Baluku et al. (2020).

 Regarding data reproducibility and availability, and with all due respect, the manuscript does include a "Data availability" section before the References, with reference [59] linking to the public repository on Figshare (DOI: 10.6084/m9.figshare.31359715) under a CC-BY 4.0 license. The repository contains three files. Underlying_Data_CDPAE.xlsx contains the 600 anonymized individual responses and the variable codebook. Extended_Data_CDPAE.xlsx includes descriptives, a correlation matrix, CFA standardized loadings, PROCESS Model 14 coefficients, bootstrap-estimated indirect effects, and tests of configural, metric, and scalar invariance by university type and gender. CDPAE_Questionnaire.xlsx contains the complete 31-item instrument with construct, dimension, and source mapping. Preprocessing (listwise with less than 2% missing data, outlier screening using Mahalanobis distance, multivariate normality using the Mardia test, and PROCESS assumptions: linearity, homoscedasticity, VIF less than 3), along with the AMOS and PROCESS syntax, are documented in Extended_Data_CDPAE.xlsx. To improve the visibility of these materials, we added a cross-reference to the repository in Section 2.3.

 Regarding the conclusions, the statements in the Abstract, Discussion, and Section 4 were reformulated as conditional associations rather than causal relationships, and the presentation of the limitations in §4.3 was strengthened. We remain open to any additional clarifications the reviewer may deem relevant.
